# A Non‐Canonical Core Transcriptional Regulatory Circuit Orchestrates Chromatin Reprogramming to Drive Osimertinib Resistance in Non‐Small Cell Lung Cancer

**DOI:** 10.1002/advs.75765

**Published:** 2026-05-20

**Authors:** Aochu Liu, Zhenguo Liu, Lizhen Jiang, Andong Huang, Xinqing Lin, Xinyue Li, Bingyuan Liu, Shiwen Hu, Qiang Pan, Junjun Huang, Liyuan Yin, Liling Jiang, Wei Yi, Wenjun Mao, Yueyuan Zheng, Xianping Shi

**Affiliations:** ^1^ Sino‐French Hoffmann Institute Guangzhou Municipal and Guangdong Provincial Key Laboratory of Protein Modification and Degradation School of Basic Medical Sciences Guangzhou Medical University Guangzhou Guangdong China; ^2^ The Affiliated Traditional Chinese Medicine Hospital Guangzhou Medical University Guangzhou Guangdong China; ^3^ Department of Thoracic Surgery The First Affiliated Hospital of Sun Yat‐Sen University Guangzhou Guangdong China; ^4^ The First Affiliated Hospital of Guangzhou Medical University National Center for Respiratory Medicine National Clinical Research Center for Respiratory Disease State Key Laboratory of Respiratory Disease Guangzhou Institute of Respiratory Health Guangzhou China; ^5^ Guangzhou Municipal and Guangdong Provincial Key Laboratory of Molecular Target & Clinical Pharmacology the NMPA and State Key Laboratory of Respiratory Disease School of Pharmaceutical Sciences Guangzhou Medical University Guangzhou China; ^6^ Lung Cancer Center West China Hospital Sichuan University Chengdu China; ^7^ Department of Cardiothoracic Surgery Wuxi Medical Center the Affiliated Wuxi People's Hospital of Nanjing Medical University Wuxi People's Hospital Nanjing Medical University Wuxi China; ^8^ Wuxi College of Clinical Medicine Nanjing Medical University Wuxi China; ^9^ Clinical Big Data Research Center Scientific Research Center The Seventh Affiliated Hospital of Sun Yat‐Sen University Shenzhen Guangdong China

**Keywords:** core regulatory circuit, ferroptosis, ID3, neuroendocrine differentiation, non‐small cell lung cancer, osimertinib resistance, super‐enhancer

## Abstract

Osimertinib resistance represents a major therapeutic challenge in non‐small cell lung cancer (NSCLC), yet the underlying epigenetic mechanisms remain incompletely understood. Through integrated epigenomic and transcriptomic profiling, we identified a resistance‐specific, non‐canonical transcriptional circuit driven by the coordinated action of three core components: ID3, SMAD3, and NR2F2. These transcription factors form protein complexes that co‐bind to shared enhancers or promoters, reciprocally enhancing each other's transcriptional activity and that of their downstream targets. Mechanistically, ID3 mediates the chromatin residence of the SMAD3‐NR2F2 complex through its non‐canonical function as a structural co‐factor. This rewires the transcriptional program of resistant cells to ultimately drive resistance via direct upregulation of HIF2A, a dual‐function hub that concurrently promotes neuroendocrine differentiation and suppresses ferroptosis. Functional studies confirmed that disrupting this circuit or inhibiting its downstream effector HIF2A restored Osimertinib sensitivity in resistant models. Our study elucidates a novel epigenetic paradigm through which a non‐canonical, self‐reinforcing transcriptional circuit co‐opts lineage plasticity and cell death evasion to drive Osimertinib resistance, thereby establishing a therapeutically actionable target for overcoming this aggressive phenotype.

## Graphical abstract

A non‐canonical core transcriptional regulatory circuit, composed of ID3, SMAD3, and NR2F2, drives Osimertinib resistance in non‐small cell lung cancer through super‐enhancer‐mediated activation of EPAS1, which couples neuroendocrine differentiation with ferroptosis evasion. Pharmacological disruption of this circuit with SIS3 or Belzutifan restores Osimertinib sensitivity, identifying a promising therapeutic strategy for resistant NSCLC.

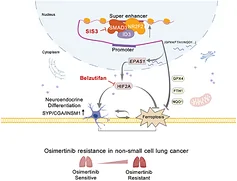

## 1 Introduction

Non‐small cell lung cancer (NSCLC) remains the leading cause of cancer‐related deaths globally [1], with a substantial proportion of cases driven by activating mutations in the epidermal growth factor receptor (EGFR) gene [2]. The development of EGFR tyrosine kinase inhibitors (EGFR‐TKIs), including first‐ and second‐generation agents such as gefitinib and afatinib, has significantly improved clinical outcomes in EGFR‐mutant NSCLC [3, 4]. Osimertinib, a third‐generation EGFR‐TKI, has become the standard first‐line therapy due to its efficacy against both sensitizing EGFR mutations and the T790M resistance mutation [5]. Owing to its improved central nervous system penetration and favorable toxicity profile, Osimertinib has become the preferred first‐line therapy for EGFR‐mutant NSCLC [6].

Acquired resistance inevitably limits Osimertinib's efficacy [7]. Multiple resistance mechanisms have been described, including secondary EGFR mutations [8, 9, 10], activation of bypass signaling pathways [8, 11, 12], and histological transformation [13]. However, these genetic alterations alone cannot fully account for the complexity and heterogeneity of resistance phenotypes. Transcriptional and epigenetic reprogramming are pivotal in sustaining drug‐tolerant states [14, 15]. Specifically, super‐enhancers (SEs), which are large enhancer clusters densely bound by coactivators, have emerged as critical regulators of cell identity and oncogenic transcription [16, 17]. SEs remodeling is a critical driver of therapeutic resistance. SE‐mediated activation of RAD23A, NR3C1, and HDAC4 promotes drug tolerance in ovarian, gastric, and lung cancers [18, 19, 20]. In NSCLC, SEs specifically induce Osimertinib resistance by driving PADI family overexpression [21].

SEs frequently regulate multiple functionally related genes, enabling the coordinated regulation of biological processes such as lineage specification, immune responses, and tumorigenesis [22, 23, 24]. This has drawn increasing attention to the core transcriptional regulatory circuit (CRC) model, in which a small clique of master transcription factors binds to their own and one another's SEs, establishing interconnected autoregulatory loops that stabilize oncogenic states [25, 26]. Such mechanisms are pivotal in lung adenocarcinoma, as seen with the E2F2‐B‐Myb‐FOXM1 circuitry driving tumor progression [27]. Similarly, in osteosarcoma, SE‐driven CRCs have been identified as key determinants of metastasis and chemoresistance, underscoring the fundamental role of these transcriptional networks in driving tumor evolution [28]. Previously, we mapped such circuits in Ewing sarcoma [29] and resistant prostate cancer [30]. Yet, emerging evidence suggests that CRCs are more structurally diverse than traditionally thought. Non‐canonical mechanisms, including mutual repression and compensatory cofactor recruitment, have been observed in multiple tumor types. For example, rhabdomyosarcoma exhibits a negative regulatory loop between MYOD1/MYOG and SOX8 [31], while in lung adenocarcinoma, SE‐driven LINC01977 recruits SMAD3 to activate ZEB1 [32]. These complexities suggest that SEs may orchestrate resistance through distinct, context‐dependent regulatory architectures that remain to be fully characterized.

Here, we identify a super‐enhancer‐driven circuit centered on the ID3‐SMAD3/NR2F2 complex as a master regulator of Osimertinib resistance. Crucially, ID3 functions as an essential scaffold, stabilizing the SMAD3/NR2F2 complex on chromatin to coordinately drive neuroendocrine differentiation and suppress ferroptosis. This non‐canonical epigenetic axis enforces a robust resistant phenotype, unveiling a novel therapeutic vulnerability for NSCLC.

## 2 Results

### 2.1 Unique Super‐Enhancers Drive Transcription Factor‐Mediated Regulation of Osimertinib Resistance in Non‐Small Cell Lung Cancer

**FIGURE 1:**
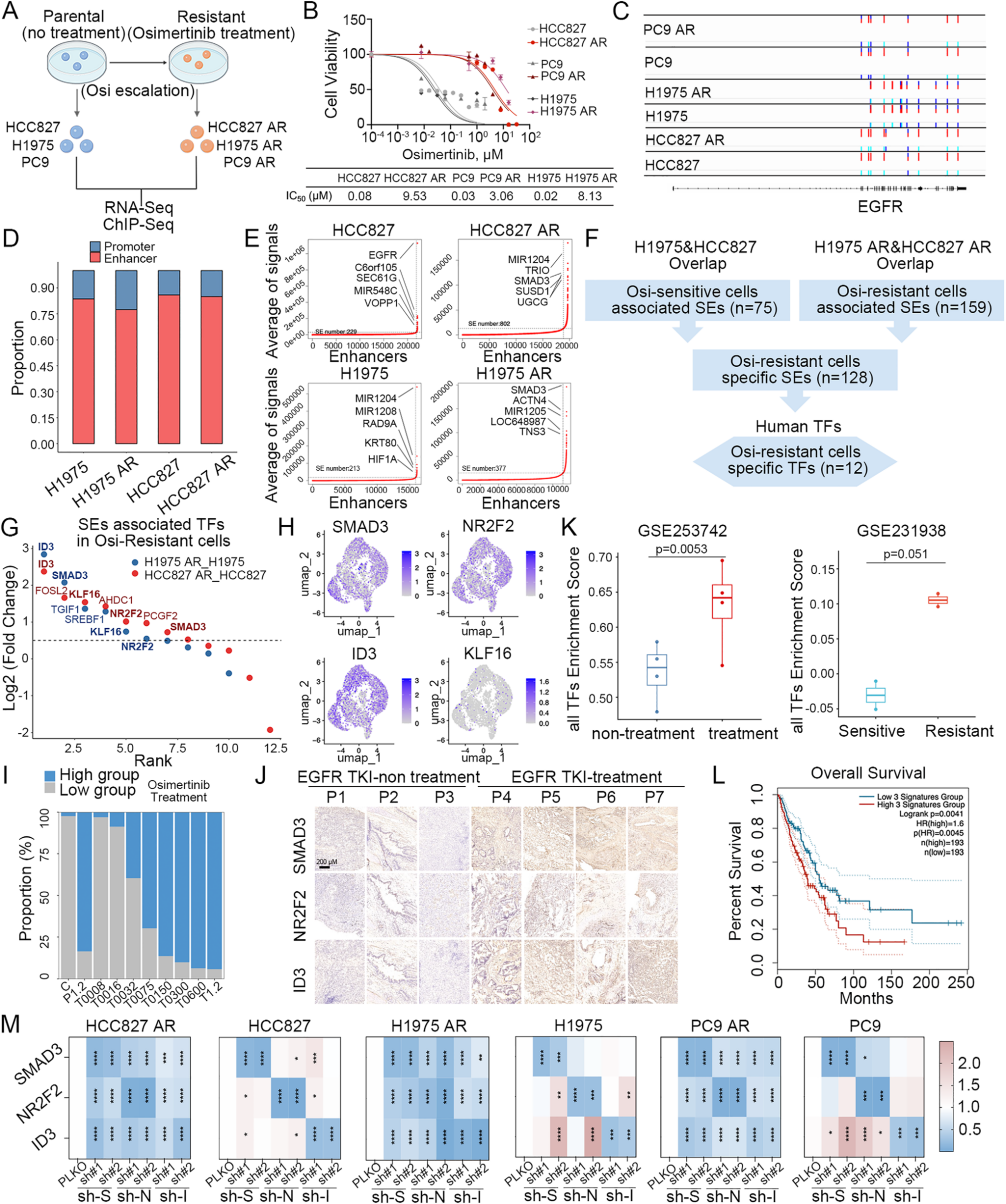
Distinct Super‐Enhancers Mediate Transcription Factor‐Driven Regulation of Osimertinib Resistance in Non‐Small Cell Lung Cancer. (a) Schematic representation of the establishment of Osimertinib‐resistant cell lines (HCC827 AR, H1975 AR, PC9 AR). (b) Dose‐response curves of parental and resistant cell lines after Osimertinib treatment. IC_50_ values for each cell line are listed in the table below. (c) Integrative Genomics Viewer (IGV) visualization of EGFR locus variants identified by whole‐exome sequencing in HCC827/HCC827 AR, H1975/H1975 AR, and PC9/PC9 AR cell line pairs. Variant tracks are displayed using the “Color by allele frequency” setting: red indicates the variant allele fraction, dark blue indicates the reference allele fraction, and cyan denotes homozygous variants. (d) Proportional distribution of H3K27ac peaks classified as promoter or enhancer regions in each cell line based on H3K27ac ChIP‐seq. **(e)** ROSE analysis for the identification of super‐enhancers (SEs) in each cell line. (f) Workflow for the screening of resistant‐cell‐specific SEs and their associated transcription factors. (g) Log2 fold change of SE‐associated transcription factors in H1975 AR vs H1975 (blue) and HCC827 AR vs HCC827 (red), ranked by expression change. The dashed line indicates the cutoff used for selecting candidate TFs. (h) UMAP plots showing the single‐cell expression distribution of candidate TFs (SMAD3, NR2F2, ID3, KLF16) in PC9 cells under Osimertinib treatment. (i) Proportion of cells with high or low expression of the ID3/SMAD3/NR2F2 module across PC9 cells treated with increasing Osimertinib concentrations. (j) Immunohistochemical (IHC) staining of SMAD3, NR2F2, and ID3 in patient tumor tissues from pre‐ and post‐Osimertinib treatment groups. (k) Comparison of the ID3/SMAD3/NR2F2 module enrichment score between EGFR‐TKI‐treated and non‐treated tumors (GSE253742, left), and between Osimertinib‐resistant and sensitive samples (GSE231938, right). (l) Kaplan‐Meier analysis comparing high and low expression groups of the combined ID3/SMAD3/NR2F2 signature in TCGA NSCLC patient data. (m) qPCR analysis of gene expression following knockdown of SMAD3, NR2F2, or ID3 in HCC827/HCC827 AR, H1975/H1975 AR, and PC9/PC9 AR cells. sh‐S, shSMAD3; sh‐N, shNR2F2; sh‐I, shID3. Data are presented as mean ± SD from three independent experiments. ^*^ *p* < 0.05; ^**^ *p* < 0.01; ^***^ *p* < 0.001; ^****^ *p* < 0.0001 (two‐tailed Student's *t*‐test).

To investigate mechanisms of resistance to Osimertinib, we generated three pairs of parental and Osimertinib‐resistant EGFR‐mutant lung cancer cell lines, HCC827/AR, H1975/AR, and PC9/AR (Figure 1a). Resistant cell lines showed >100‐fold higher IC_50_ compared to their parental counterparts (Figure 1b). Resistant and sensitive cells exhibit distinct morphological differences, with the resistant cells demonstrating more prominent neuroendocrine characteristics (Figure S1a–c). To rigorously validate their authenticity, we conducted systematic comparative analyses. Exome sequencing, RNA‐Seq, and ChIP‐Seq analyses were performed, and resistance‐associated signatures identified in resistant cells are presented. (Figure S1d–g). Additionally, Osimertinib‐resistant cell lines did not exhibit any additional classical EGFR mutations. (Figure 1c) [33, 34].

We next performed H3K27ac ChIP–Seq in HCC827, HCC827 AR, H1975, and H1975 AR cells to map active regulatory elements, including promoters and enhancers, and defined the SE landscapes for each cell line (Figure 1d,e). This analysis revealed 128 SE‐associated genes specifically in Osimertinib‐resistant cells, among which 12 were transcription factors (Figure 1f). Integrated analysis with RNA‐seq data revealed that four of these transcription factors, including ID3, SMAD3, NR2F2, and KLF16, were consistently upregulated in the resistant cells (Figure 1g).

We further evaluated the role of these factors using single‐cell transcriptomic data from PC9 cells under acute or continuous Osimertinib exposure [35]. All three genes were consistently upregulated upon treatment, whereas KLF16 expression remained undetectable under all conditions (Figure 1h; Figure S2a). Interestingly, when evaluated as a gene module, the combined expression of ID3, SMAD3, and NR2F2 was markedly increased under both treatment regimens, suggesting cooperative regulation of a resistance‐associated transcriptional program (Figure 1i). Consistent with this, elevated expression of ID3, SMAD3, and NR2F2 was observed in tumor tissues from patients following EGFR‐TKI treatment (Figure 1j). Additionally, increased expression of this gene module was detected in patient‐derived tumor samples exposed to EGFR‐TKI and in resistant states, highlighting its clinical relevance (Figure 1k; Figure S2b). To corroborate these findings in a clinical context, we analyzed tumor samples from patients pre‐ and post‐EGFR‐TKI treatment. H3K27ac ChIP–Seq on isolated tumor cells revealed markedly elevated signals at the ID3, SMAD3, and NR2F2 loci in treatment samples (Figure S2c).

Furthermore, TCGA analysis revealed that high co‐expression of ID3, SMAD3, and NR2F2, as opposed to high expression of individual factors alone, correlated with poorer prognosis and greater malignancy in non‐small cell lung cancer (Figure 1l; Figure S2d). To investigate the regulatory interactions among three transcription factors, we individually knocked down each transcription factor in HCC827 AR, H1975 AR, and PC9 AR cells. Silencing one factor resulted in a concomitant decrease in the expression of the other two, suggesting the existence of a positive feedback regulatory loop (Figure 1m; Figure S2e,f).

### 2.2 ID3 Acts as a Transcriptional Co‐Activator by Binding SMAD3 and NR2F2 to Stabilize Their DNA‐Binding Affinity and Enhance Target Gene Transcription

**FIGURE 2:**
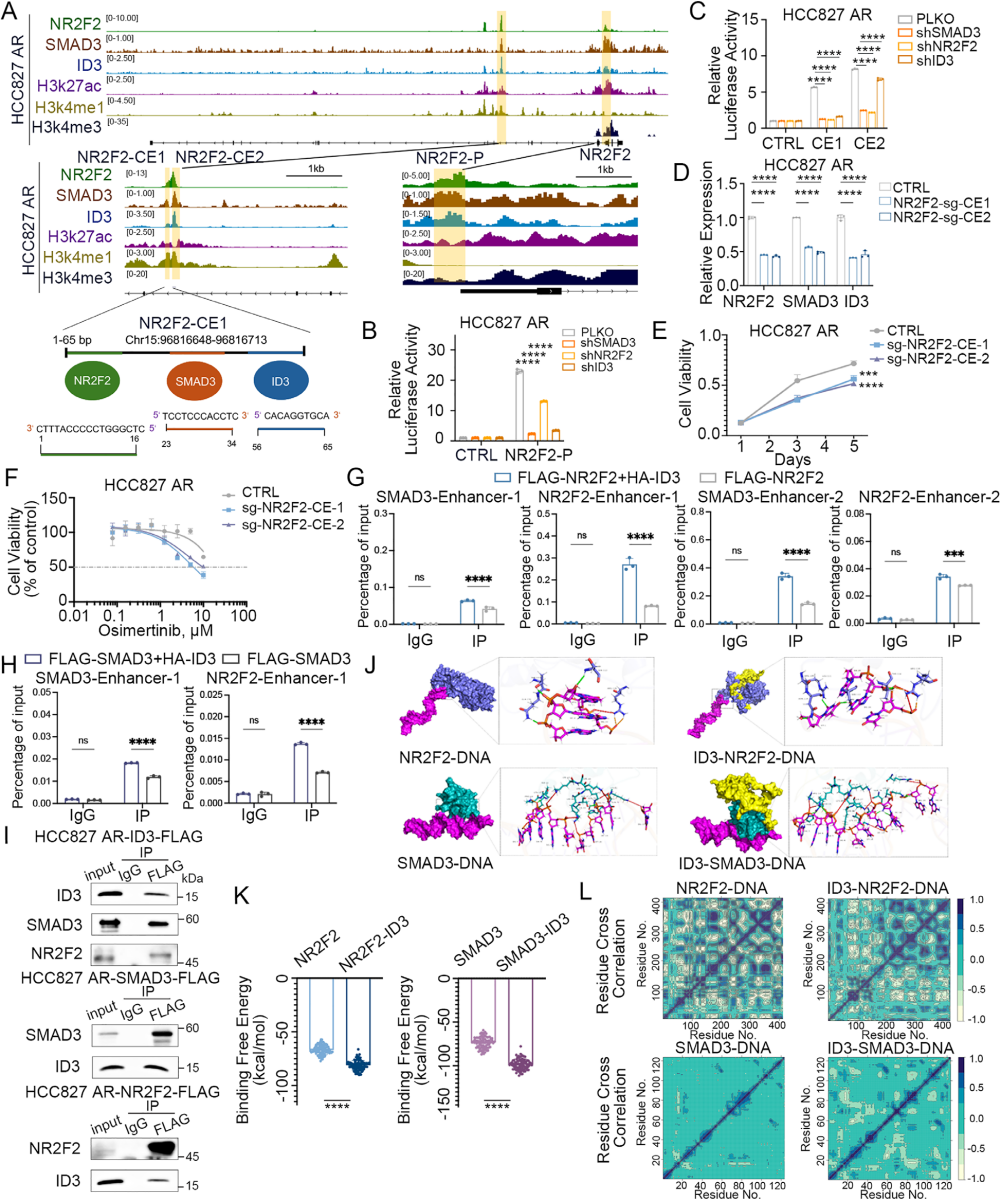
ID3 Functions as a Transcriptional Co‐Activator by Enhancing DNA‐Binding Affinity of SMAD3 and NR2F2, Facilitating Target Gene Activation. (a) ChIP‐seq enrichment profiles of NR2F2, SMAD3, ID3, H3K27ac, H3K4me1, and H3K4me3 at the *NR2F2* locus in HCC827 AR cells. Candidate cis‐regulatory elements (NR2F2‐CE1, NR2F2‐CE2, NR2F2‐P) are indicated. Bottom: schematic of NR2F2‐CE1 with predicted NR2F2, SMAD3, and ID3 binding motifs. (b, c) Luciferase reporter assays of NR2F2‐P (b), NR2F2‐CE1, and NR2F2‐CE2 (c) activity following knockdown of SMAD3, NR2F2, or ID3 in HCC827 AR cells. (d) qPCR of *NR2F2*, *SMAD3*, and *ID3* in HCC827 AR cells following KRAB–dCas9‐mediated repression of NR2F2‐CE1 (sg‐CE1) or NR2F2‐CE2 (sg‐CE2). (e, f) Cell viability (e) and Osimertinib dose‐response (f) of HCC827 AR cells with CE1 or CE2 repression. (g, h) ChIP‐qPCR of FLAG‐NR2F2 (g) and FLAG‐SMAD3 (h) occupancy at the indicated enhancers, with or without HA‐ID3 co‐expression. (i) Co‐immunoprecipitation assays demonstrating pairwise interactions among ID3, SMAD3, and NR2F2 in HCC827 AR cells. (j) Structural snapshots from molecular dynamics (MD) simulations of NR2F2–DNA, SMAD3–DNA, ID3–NR2F2–DNA, and ID3–SMAD3–DNA complexes at NR2F2‐CE1. (k) Binding free energy of NR2F2–DNA and SMAD3–DNA complexes ± ID3, from MD trajectories. (l) Dynamic cross‐correlation maps (DCCM) of the corresponding complexes. Data are presented as mean ± SD from three independent experiments. ns, not significant; ^*^ *p* < 0.05; ^**^ *p* < 0.01; ^***^ *p* < 0.001; ^****^ *p* < 0.0001 (two‐tailed Student's *t*‐test).

ChIP–seq analysis revealed co‐occupancy of ID3, SMAD3, and NR2F2 at their respective enhancer regions, with binding sites confined within a single nucleosome span (Figure 2a; Figure S3a,b). Knockdown of ID3, SMAD3, and NR2F2 significantly suppressed the transcriptional activity of both the promoter and enhancer regions of SMAD3, NR2F2, and ID3 (Figure 2b,c; Figure S3c–f). Moreover, targeted repression of the co‐occupied enhancer near the NR2F2 and ID3 locus using a KRAB‐dCas9 system downregulated all three transcription factors, resulting in reduced proliferation of HCC827 AR cells and increased sensitivity to Osimertinib (Figure 2d–f; Figure S3g–i).

Since ID3 lacks an intrinsic DNA‐binding domain, we investigated whether it modulates the chromatin occupancy of its transcriptional partners, SMAD3 and NR2F2. ChIP‐qPCR results revealed that overexpression of ID3 markedly enhanced the enrichment of both SMAD3 and NR2F2 at key regulatory loci, including their own enhancers and the promoters of ID3, SMAD3, and NR2F2 (Figure 2g–h; Figure S3j). Co‐immunoprecipitation assays revealed that ID3 physically interacts with both SMAD3 and NR2F2 (Figure 2i). To investigate whether this interaction enhances DNA‐binding stability, we performed molecular dynamics simulations of SMAD3 and NR2F2 bound to the NR2F2‐CE1 site in the presence or absence of ID3. These simulations revealed that ID3 significantly stabilizes the SMAD3‐DNA and NR2F2‐DNA complex throughout the dynamic process, as supported by analyses of binding free energy, interaction energy, and dynamic cross‐correlation maps (DCCM) (Figure 2j–l; Figure S3k–n). Moreover, the interaction with ID3 led to a marked increase in the number of chemical bonds involved in the DNA‐binding interface. Specifically, upon ID3 binding, the number of chemical bonds between SMAD3 and DNA increased from 14 to 20, while the number of bonds between NR2F2 and DNA rose from 6 to 9 (Figure 2j; Table S1). These findings further underscore the profound stabilization of the DNA‐binding complex, highlighting the critical role of ID3 in modulating the interaction dynamics.

Further insights were gained from principal component analysis (PCA) (Figure S3o), which revealed that ID3 binding induced conformational changes in both SMAD3 and NR2F2. These structural alterations were accompanied by shifts in the chemical bond sites involved in the protein‐DNA interactions (Table S2), suggesting that the enhanced DNA‐binding stability observed upon ID3 binding is mediated, at least in part, through these conformational changes. This remodeling modulates the interaction landscape and facilitates a more stable binding of both SMAD3 and NR2F2 to the DNA.

### 2.3 ID3, SMAD3, and NR2F2 Synergistically Regulate the Transcriptional Program of Osimertinib‐Resistant Cells

**FIGURE 3:**
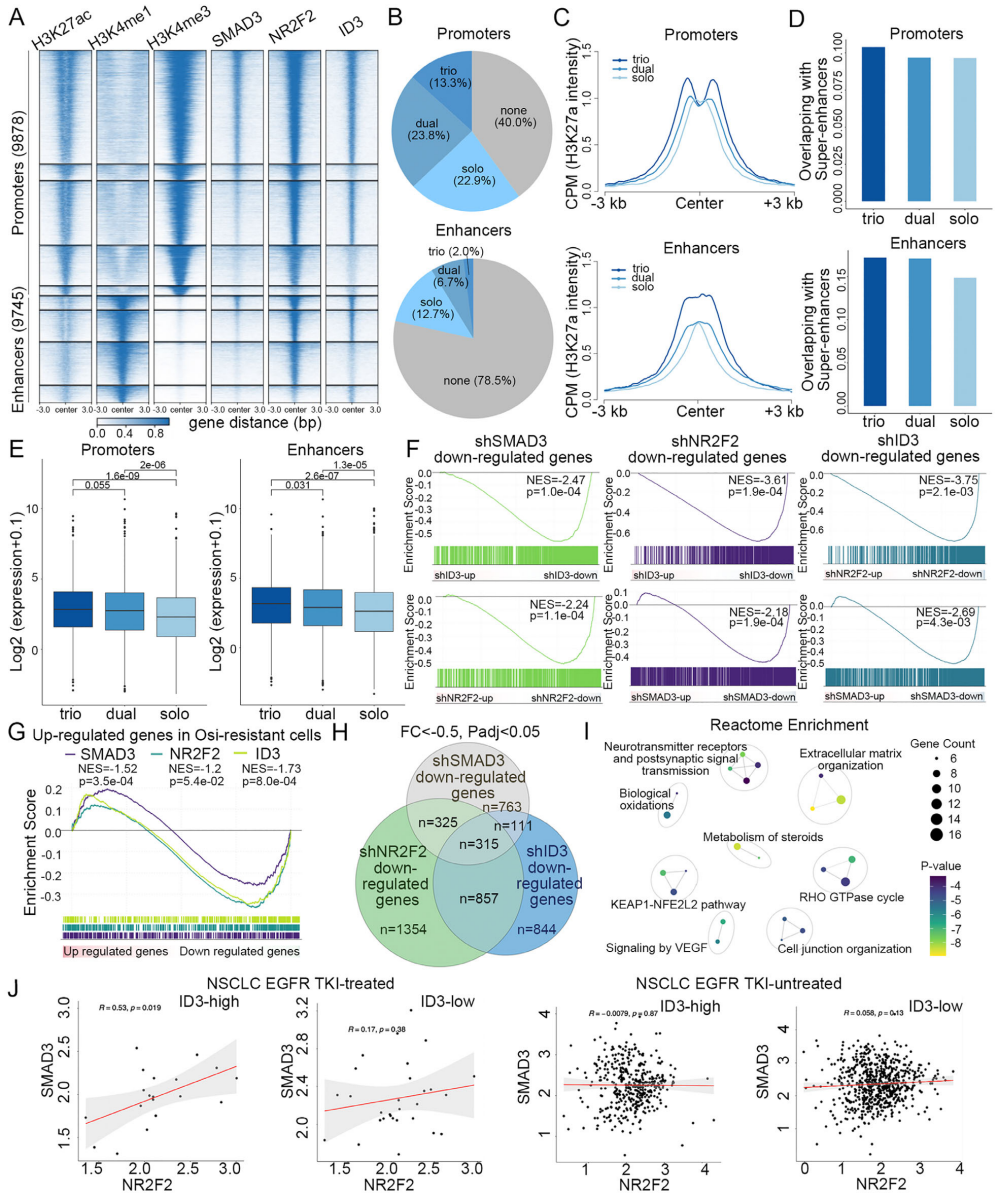
ID3, SMAD3, and NR2F2 Synergistically Regulate the Osimertinib‐Resistance Transcriptional Program. (a) Heatmaps showing H3K27ac, H3K4me1, H3K4me3, SMAD3, NR2F2, and ID3 enrichment at promoter (*n* = 9,878) and enhancer (*n* = 9,745) regions in HCC827 AR cells, based on ChIP‐seq and CUT&Tag data. (b) Pie charts showing the distribution of binding patterns at promoters and enhancers: trio (co‐bound by ID3, SMAD3, and NR2F2), dual, solo, and none. (c) Average H3K27ac signal intensity profiles (CPM) at promoters and enhancers across trio, dual, and solo groups. (d) Proportion of trio, dual, and solo regions overlapping with super‐enhancers at promoters and enhancers. (e) Box plots comparing expression levels of genes associated with trio, dual, and solo binding at promoters and enhancers. (f) GSEA showing reciprocal enrichment of down‐regulated genes from each knockdown (shSMAD3, shNR2F2, shID3) in the ranked gene lists of the other two knockdowns in HCC827 AR cells. (g) GSEA showing enrichment of ID3, SMAD3, and NR2F2 positively regulated target genes in genes differentially expressed between Osimertinib‐resistant and parental cells. (h) Venn diagram showing the overlap of down‐regulated genes following shSMAD3, shNR2F2, or shID3 in HCC827 AR cells. (i) Reactome pathway enrichment analysis of the co‐regulated genes shared by all three knockdowns. (j) Correlation between SMAD3 and NR2F2 expression in ID3‐high and ID3‐low subgroups of EGFR‐TKI‐treated and untreated NSCLC patient samples. Box plots in (e) show the median (center line), interquartile range (box), and 1.5× IQR (whiskers); statistical significance was assessed by two‐sided Wilcoxon rank‐sum test, with *p*‐values indicated above each comparison. GSEA in (f, g) was performed using the GSEA preranked algorithm with 1,000 permutations; normalized enrichment scores (NES) and nominal *p*‐values are shown. Reactome pathway enrichment in (i) was performed using a hypergeometric test, with *p*‐values shown by the color scale and gene counts by bubble size. Correlations in (j) were assessed by two‐sided Pearson correlation; correlation coefficients (R) and *p*‐values are indicated. Differentially expressed genes (RNA‐seq) were defined as |log_2_FC| > 0.5 and adjusted *p* < 0.05 (DESeq2, Benjamini–Hochberg correction).

To elucidate the transcriptional regulatory mechanisms of ID3, SMAD3, and NR2F2 in Osimertinib‐resistant cells, we integrated CUT&Tag data with histone modification profiles (H3K4me1, H3K4me3, and H3K27ac) in HCC827 AR cells, to map their binding landscapes at promoters and enhancers (Figure 3a). Based on occupancy patterns, genomic regions were classified into four categories— ‘trio’, ‘dual’, ‘solo’, and ‘none’, which represent binding by all three factors, any two, one, or none, respectively; their distributions across promoter and enhancer regions were determined (Figure 3b). Notably, regions in the ‘trio’ group exhibited significantly higher H3K27ac enrichment than other groups, indicating enhanced chromatin accessibility and transcriptional activity (Figure 3c), and were more frequently associated with annotated super‐enhancers, underscoring the functional synergy of concurrent transcription factor binding (Figure 3d). Correspondingly, gene expression levels were markedly elevated in the ‘trio’ group compared with other groups for both promoter‐ and enhancer‐associated genes, supporting that co‐binding by these factors synergistically promotes transcriptional activation (Figure 3e).

RNA‐Seq analysis following individual knockdown of ID3, SMAD3, and NR2F2 revealed that each factor regulates a distinct set of genes (Figure S4a). Gene Set Enrichment Analysis (GSEA) of the downregulated gene sets demonstrated significant overlap among all three factors (Figure 3f), a trend also observed among the upregulated gene sets (Figure S4b). Furthermore, comparison of upregulated genes in HCC827 AR and H1975 AR cells relative to their parental lines identified a subset significantly enriched among the targets positively regulated by ID3, SMAD3, and NR2F2 (Figure 3g). These findings highlight the strong similarity in the transcriptional programs governed by these three factors, underscoring their potential roles in Osimertinib resistance.

To further elucidate how ID3, SMAD3, and NR2F2 contribute to acquired Osimertinib resistance in non‐small cell lung cancer, we intersected the gene sets positively regulated by all three factors. These shared targets were significantly enriched among genes upregulated in resistant cells (Figure 3h; Figure S4c), implicating them as key mediators of the resistant phenotype. Although EGFR itself was ruled out, pathway enrichment analysis revealed significant overrepresentation of neurotransmitter receptors and postsynaptic signaling pathways, as well as the KEAP1‐NFE2L2 axis. This finding is consistent with the pathways enriched in genes highly expressed in the drug‐resistant cells, implicating these processes as potential mechanisms underlying Osimertinib resistance (Figure 3i; Figure S4d,e). Given the transcriptional co‐activating function of ID3, we next examined patient samples before and after EGFR‐TKI treatment. In treatment‐naïve samples, SMAD3 and NR2F2 expression showed no correlation regardless of ID3 levels. In contrast, in post‐treatment samples, high ID3 expression was associated with a markedly stronger correlation between SMAD3 and NR2F2, with correlation coefficients exceeding those observed in samples with low ID3 expression (Figure 3j). These results suggest that ID3 facilitates the cooperative activity of SMAD3 and NR2F2 in the context of EGFR‐TKI treatment.

### 2.4 ID3, SMAD3, and NR2F2 Cooperatively Promote the Malignant Phenotype of Osimertinib‐Resistant Non‐Small Cell Lung Cancer

**FIGURE 4:**
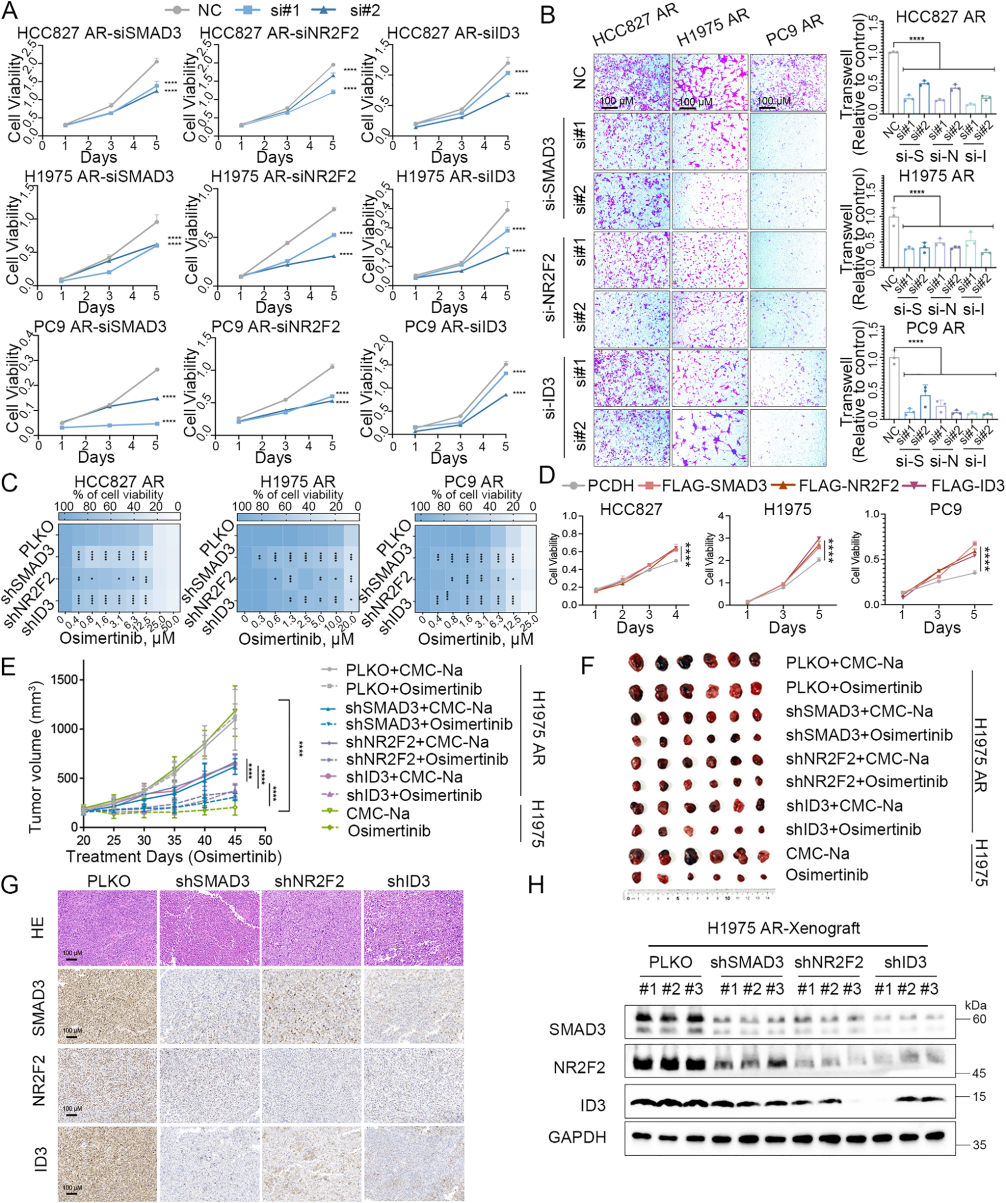
ID3, SMAD3, and NR2F2 Cooperatively Drive the Malignant Phenotype of Osimertinib‐Resistant NSCLC. (a) Cell viability curves of HCC827 AR, H1975 AR, and PC9 AR cells following siRNA‐mediated knockdown of SMAD3, NR2F2, or ID3. (b) Transwell migration assays in HCC827 AR, H1975 AR, and PC9 AR cells following siRNA knockdown of SMAD3 (si‐S), NR2F2 (si‐N), or ID3 (si‐I); representative images (left) and quantification relative to NC control (right). (c) Heatmaps showing percent cell viability of HCC827 AR, H1975 AR, and PC9 AR cells across a range of Osimertinib concentrations following stable shRNA knockdown of SMAD3 (shSMAD3), NR2F2 (shNR2F2), or ID3 (shID3); PLKO as vector control. (d) Cell viability curves of parental HCC827, H1975, and PC9 cells overexpressing FLAG‐SMAD3, FLAG‐NR2F2, or FLAG‐ID3; PCDH as empty vector control. (e, f) Subcutaneous xenografts in nude mice using parental H1975 cells or H1975 AR cells stably transduced with PLKO, shSMAD3, shNR2F2, or shID3. (e) Tumor growth curves; (f) representative gross images of resected tumors at endpoint. (g) H&E staining and IHC staining for SMAD3, NR2F2, and ID3 in xenograft tumor tissues from H1975 AR groups. (h) Western blot analysis of SMAD3, NR2F2, and ID3 protein levels in three independent xenograft tumors per group. Data are presented as mean ± SD from *n* = 3 independent experiments, except (e) presented as mean ± SEM (*n* = 6 mice per group). Statistical significance was assessed by two‐way ANOVA with Tukey's post hoc test (a, d, e), one‐way ANOVA with Dunnett's post hoc test versus NC (b), or two‐tailed unpaired Student's *t*‐test versus PLKO at each concentration (c). ns, not significant; ^*^ *p* < 0.05; ^**^ *p* < 0.01; ^***^ *p* < 0.001; ^****^ *p* < 0.0001.

To investigate the functional roles of ID3, SMAD3, and NR2F2 in Osimertinib‐resistant NSCLC, we performed loss‐of‐function experiments in multiple resistant cell lines (HCC827 AR, H1975 AR, and PC9 AR). Knockdown of any of these three factors significantly suppressed cell proliferation and migration and enhanced sensitivity to Osimertinib (Figure 4a–c; Figure S5a). Conversely, overexpression of these factors in parental HCC827, H1975, and PC9 cells enhanced proliferative and migratory capacities and reduced drug sensitivity (Figure 4d; Figure S5b–d). These results demonstrate that all three factors contribute critically to the malignant behavior of Osimertinib‐resistant NSCLC cells.

**FIGURE 5:**
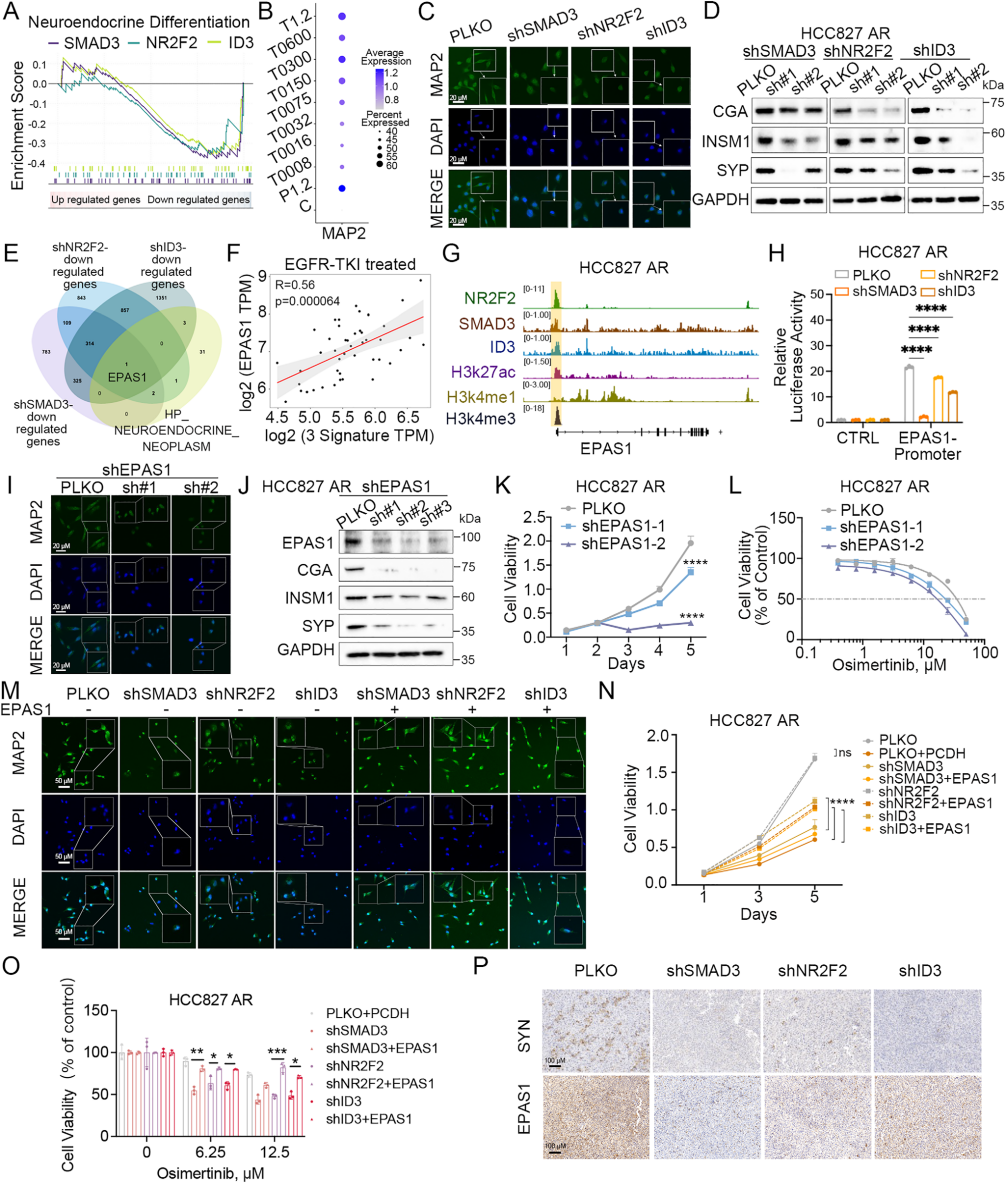
ID3, SMAD3, and NR2F2 Promote Neuroendocrine Differentiation Driving Osimertinib Resistance in NSCLC. (a) GSEA showing enrichment of a neuroendocrine differentiation gene set among genes downregulated upon shSMAD3, shNR2F2, or shID3 in HCC827 AR cells. (b) Dot plot showing *MAP2* expression in PC9 cells across increasing Osimertinib concentrations from single‐cell RNA‐seq data (GSE247684); dot size indicates the percentage of cells expressing *MAP2*, color indicates average expression. (c) Immunofluorescence staining of MAP2 (green) in H1975 AR cells following shRNA knockdown of SMAD3, NR2F2, or ID3; DAPI (blue) marks nuclei. (d) Western blot of CGA, INSM1, and SYP in HCC827 AR cells following knockdown of SMAD3, NR2F2, or ID3 with two independent shRNAs. (e) Venn diagram showing overlap among down‐regulated genes following shSMAD3, shNR2F2, or shID3 and the HP_NEUROENDOCRINE_NEOPLASM gene set. (f) Pearson correlation between *EPAS1* expression and the combined ID3/SMAD3/NR2F2 signature in EGFR‐TKI‐treated NSCLC patients (TCGA). (g) ChIP‐seq and CUT&Tag tracks showing NR2F2, SMAD3, ID3, H3K27ac, H3K4me1, and H3K4me3 enrichment at the *EPAS1* locus in HCC827 AR cells. (h) Luciferase reporter assay of *EPAS1* promoter activity in HCC827 AR cells following shRNA knockdown of SMAD3, NR2F2, or ID3. (i) Immunofluorescence staining of MAP2 in H1975 AR cells following EPAS1 knockdown. (j) Western blot of EPAS1, CGA, INSM1, and SYP in HCC827 AR cells following EPAS1 knockdown. (k, l) Cell viability (k) and Osimertinib dose‐response (l) of HCC827 AR cells with EPAS1 knockdown. (m–o) Rescue experiments in HCC827 AR cells with shSMAD3, shNR2F2, or shID3, with or without EPAS1 overexpression, (m) MAP2 immunofluorescence; (n) cell viability; (o) cell viability across Osimertinib concentrations. (p) IHC staining of SYN and EPAS1 in xenograft tumors from H1975 AR cells with shSMAD3, shNR2F2, or shID3. Data are presented as mean ± SD from *n* = 3 independent experiments. Statistical significance was assessed by one‐way ANOVA with Dunnett's post hoc test versus PLKO (h), two‐way ANOVA with Tukey's post hoc test (k, l, n, o). ^*^ *p* < 0.05; ^**^ *p* < 0.01; ^***^ *p* < 0.001; ^****^ *p* < 0.0001.

We further validated these findings in vivo using mouse xenograft models. The proliferation rate of H1975 cells was comparable to that of H1975 AR cells. However, treatment with Osimertinib significantly inhibited the proliferation of H1975 cells, whereas no similar inhibitory effect was observed in H1975 AR cells. Silencing SMAD3, NR2F2, or ID3 in H1975 AR cells substantially delayed tumor growth. Subsequent oral administration of Osimertinib further suppressed tumor progression compared with the CMC–Na control group. Notably, knockdown of these factors strongly potentiated the antitumor efficacy of Osimertinib in H1975 AR‐derived xenografts (Figure 4e,f; Figure S5e). Gene expression analysis of tumor tissues further supported a positive regulatory relationship among ID3, SMAD3, and NR2F2 (Figure 4g,h), consistent with their cooperative role in driving tumorigenesis.

To directly assess the functional synergy among these factors, we performed combinatorial knockdown experiments in HCC827 AR cells. Simultaneous suppression of any two factors resulted in significantly stronger inhibition of cell proliferation compared to individual knockdown (Figure S5f,g). Together, these data indicate that ID3, SMAD3, and NR2F2 act cooperatively to promote the malignant phenotype and Osimertinib resistance in NSCLC.

To determine whether the CRC factors are causally required for the acquisition of Osimertinib resistance, we performed loss‐ and gain‐of‐function escape assays in parental sensitive cells. Knockdown of any one of ID3, SMAD3, or NR2F2 had only modest effects on colony formation in drug‐free conditions, but markedly suppressed colony formation in parental H1975 and HCC827 cells under Osimertinib treatment (Figure S6a). When subjected to stepwise Osimertinib dose escalation over 8 weeks, control PLKO cells progressively acquired resistance with rising IC_50_ values, whereas knockdown of any CRC factor substantially attenuated this increase (Figure S6b) and rendered cells largely sensitive at the end of escalation (Figure S6c). Reciprocally, ectopic overexpression of ID3, SMAD3, or NR2F2 in parental HCC827 cells significantly enhanced colony formation under Osimertinib treatment (Figure S6d). Together, these results indicate that the CRC factors are individually required for the efficient establishment of Osimertinib resistance, and that their forced expression is sufficient to partially confer a resistant phenotype in otherwise sensitive cells.

### 2.5 ID3, SMAD3, and NR2F2 Mediate Osimertinib Resistance by Promoting Neuroendocrine Differentiation in Non‐Small Cell Lung Cancer Cells

Based on pathway enrichment analysis of prior sequencing data, GSEA revealed significant enrichment of neuroendocrine differentiation‐associated gene sets among the targets regulated by ID3, SMAD3, and NR2F2 (Figure 5a). Consistent with this, Osimertinib‐resistant cells displayed enhanced neuroendocrine features (Figure S1a–c), and MAP2 expression was markedly upregulated in PC9 cells following Osimertinib treatment (Figure 5b). Knockdown of ID3, SMAD3, and NR2F2 in HCC827 AR and H1975 AR cells significantly suppressed the neuroendocrine phenotype (Figure 5c,d; Figure S7a,b). Overexpression of the three transcription factors in H1975 cells resulted in an enhanced neuroendocrine phenotype (Figure S7c).

Notably, we identified EPAS1 (HIF‐2α), a known driver of neuroendocrine differentiation, angiogenesis, and metabolic adaptation, as a common downstream target of all three transcription factors (Figure 5e). In EGFR‐TKI‐treated NSCLC patient samples, the positive correlation among ID3, SMAD3, NR2F2, and EPAS1 was significantly stronger than in treatment‐naïve samples (Figure 5f; Figure S7d), supporting their clinical relevance. Upon sorting the MAP2 high and low expression cell populations, it was found that during Osimertinib treatment, EPAS1 expression gradually increased and was predominantly distributed in the MAP2 high expression cell population (Figure S7e).

CUT&Tag sequencing revealed that ID3, SMAD3, and NR2F2 co‐occupy the EPAS1 promoter region (Figure 5g). Knockdown of all three factors markedly reduced EPAS1 promoter activity, indicating their essential role in its transcriptional activation (Figure 5h). Silencing EPAS1 in HCC827 AR and H1975 AR cells significantly suppressed the neuroendocrine phenotype, reduced cell proliferation, and increased sensitivity to Osimertinib (Figure 5i–l; Figure S7f,g). Conversely, EPAS1 overexpression effectively reversed the suppression of neuroendocrine features, restored proliferative capacity, and rescued Osimertinib resistance induced by ID3, SMAD3, and NR2F2 depletion (Figure 5m–o). The expression of SYN and EPAS1 in tumor tissues from mice was also significantly reduced in the transcription factor knockdown group (Figure 5p). Together, these findings demonstrate that ID3, SMAD3, and NR2F2 promote neuroendocrine differentiation and Osimertinib resistance in NSCLC cells via transcriptional activation of EPAS1.

### 2.6 ID3, SMAD3, and NR2F2 Cooperatively Suppress Ferroptosis to Promote Osimertinib Resistance in NSCLC

**FIGURE 6:**
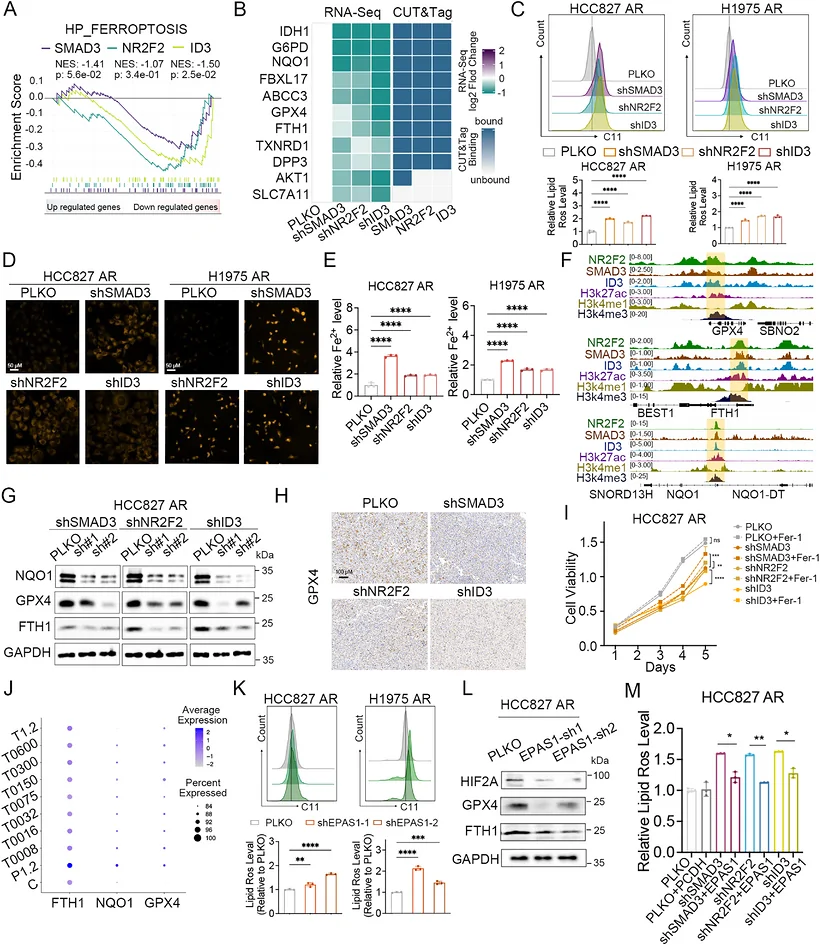
ID3, SMAD3, and NR2F2 Suppress Ferroptosis to Drive Osimertinib Resistance in NSCLC. (a) GSEA showing enrichment of the HP_FERROPTOSIS gene set among genes downregulated upon shSMAD3, shNR2F2, or shID3 in HCC827 AR cells. (b) Combined heatmap of ferroptosis‐related gene expression (RNA‐seq, left) and SMAD3/NR2F2/ID3 promoter occupancy (CUT&Tag, right) in HCC827 AR cells following the indicated knockdowns. (c) Flow cytometry analysis of lipid peroxidation (C11‐BODIPY) in HCC827 AR and H1975 AR cells following shRNA knockdown of SMAD3, NR2F2, or ID3; representative histograms (top) and quantification of relative lipid ROS levels (bottom). (d) FerroOrange staining of intracellular Fe^2^^+^ in HCC827 AR and H1975 AR cells following knockdown of SMAD3, NR2F2, or ID3. (e) Intracellular Fe^2^^+^ levels in HCC827 AR and H1975 AR cells following knockdown of SMAD3, NR2F2, or ID3, measured by colorimetric iron assay. (f) ChIP‐seq and CUT&Tag tracks showing NR2F2, SMAD3, ID3, H3K27ac, H3K4me1, and H3K4me3 enrichment at the *GPX4*, *FTH1*, and *NQO1* loci in HCC827 AR cells. (g) Western blot of NQO1, GPX4, and FTH1 in HCC827 AR cells following knockdown of SMAD3, NR2F2, or ID3 with two independent shRNAs. (h) IHC staining of GPX4 in H1975 AR xenograft tumors from PLKO, shSMAD3, shNR2F2, and shID3 groups. (i) Cell viability of HCC827 AR cells with shSMAD3, shNR2F2, or shID3, with or without Ferrostatin‐1 (Fer‐1) treatment. (j) Dot plot showing *FTH1*, *NQO1*, and *GPX4* expression in MAP2‐high PC9 cell populations across increasing Osimertinib concentrations, from single‐cell RNA‐seq data (GSE247684). (k) Flow cytometry analysis of lipid peroxidation (C11‐BODIPY) in HCC827 AR and H1975 AR cells following EPAS1 knockdown; representative histograms (top) and quantification (bottom). (l) Western blot of HIF2A, GPX4, and FTH1 in HCC827 AR cells following EPAS1 knockdown. (m) Quantification of lipid peroxidation (C11‐BODIPY) in HCC827 AR cells with shSMAD3, shNR2F2, or shID3, with or without EPAS1 overexpression. Data are presented as mean ± SD from *n* = 3 independent experiments. Statistical significance was assessed by one‐way ANOVA with Dunnett's post hoc test versus PLKO (c, e, k), one‐way ANOVA with Tukey's post hoc test(m), or two‐way ANOVA with Tukey's post hoc test (i). ^*^ *p* < 0.05; ^**^ *p* < 0.01; ^***^ *p* < 0.001; ^****^ *p* < 0.0001.

Since the KEAP1‐NFE2L2 axis is a well‐established master regulator of ferroptosis [36, 37], with its downstream effectors closely linked to ferroptosis resistance, we first investigated whether ID3, SMAD3, and NR2F2 are involved in this program. Pathway enrichment analysis of the downregulated genes upon knockdown of these factors revealed significant enrichment of the KEAP1‐NFE2L2 signaling pathway and the ferroptosis pathway among their positively regulated targets (Figure 6a). Integrated RNA‐Seq and CUT&Tag analyses further demonstrated that ID3, SMAD3, and NR2F2 occupy the regulatory regions of multiple anti‐ferroptotic effectors within the NRF2‐associated antioxidant and iron‐handling program and positively regulate their expression (Figure 6b). Consistent with this, upon knockdown of ID3, SMAD3, and NR2F2, lipid peroxidation levels and ferrous ion content increased, indicating enhanced ferroptosis, while the expression of key ferroptosis‐related genes was downregulated (Figure 6c–g). Treatment with the ferroptosis inhibitor Ferrostatin‐1 partially rescued the proliferation inhibition caused by knockdown of ID3, SMAD3, and NR2F2, and attenuated the enhanced Osimertinib sensitivity observed upon CRC factor depletion (Figure 6h,i; Figure S7h).

We next explored the potential crosstalk between the two major downstream pathways co‐regulated by these factors: neuroendocrine differentiation and ferroptosis. In MAP2‐high cell populations, Osimertinib stimulation significantly upregulated key anti‐ferroptosis molecules such as GPX4, FTH1, and NQO1, suggesting a functional interplay between neuroendocrine features and ferroptosis resistance (Figure 6j). Knockdown of EPAS1, a neuroendocrine regulator and common target of ID3/SMAD3/NR2F2, not only promoted ferroptosis but also reduced GPX4 and FTH1 expression (Figure 6k,l). Conversely, EPAS1 overexpression reversed the ferroptosis phenotype induced by ID3, SMAD3, or NR2F2 silencing (Figure 6m). Importantly, ferroptosis inhibition also attenuated the loss of the neuroendocrine phenotype caused by knockdown of these transcription factors (Figure S7i), indicating a bidirectional regulatory relationship between ferroptosis and neuroendocrine differentiation. Together, these results demonstrate that ID3, SMAD3, and NR2F2 cooperatively inhibit ferroptosis and promote Osimertinib resistance, in part through the activation of NRF2‐dependent genes and via functional interplay with neuroendocrine differentiation. EPAS1 serves as a critical hub mediating this crosstalk, underscoring its central role in the transcriptional network governing drug resistance.

### 2.7 Pharmacological Inhibition of SMAD3 and EPAS1 Suppresses the Viability of Osimertinib‐Resistant Cells

**FIGURE 7:**
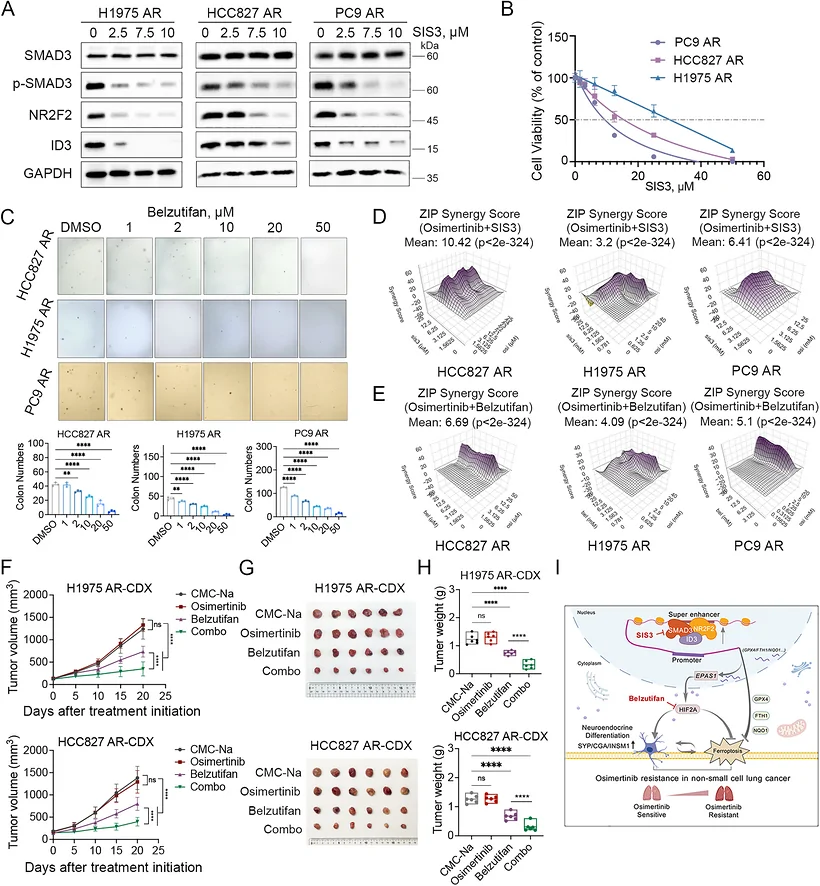
Pharmacological Inhibition of SMAD3 and EPAS1 Reduces Viability of Osimertinib‐Resistant Cells. (a) Western blot of SMAD3, phospho‐SMAD3 (p‐SMAD3), NR2F2, and ID3 in H1975 AR, HCC827 AR, and PC9 AR cells treated with the indicated concentrations of SIS3. (b) Cell viability of H1975 AR, HCC827 AR, and PC9 AR cells treated with increasing concentrations of SIS3. (c) Colony formation assays in HCC827 AR, H1975 AR, and PC9 AR cells treated with the indicated concentrations of Belzutifan; representative images (top) and quantification of colony numbers (bottom). (d, e) Synergistic effect of (d) Osimertinib combined with SIS3 and (e) Osimertinib combined with Belzutifan in HCC827 AR, H1975 AR, and PC9 AR cells. (f) Tumor growth curves of H1975 AR (top) and HCC827 AR (bottom) cell‐derived xenografts (CDX) in nude mice treated with CMC‐Na vehicle, Osimertinib, Belzutifan, or the Osimertinib+Belzutifan combination (Combo). (g) Representative gross images of resected tumors from each treatment group at endpoint. (h) Tumor weights at endpoint, quantified from (g). (i) Schematic representation of the experimental model. Data in (b, c) are presented as mean ± SD from *n* = 3 independent experiments. Data in (f, h) are presented as mean ± SEM (*n* = 6 mice per group). Statistical significance was assessed by one‐way ANOVA with Dunnett's post hoc test versus DMSO (b, c), or two‐way ANOVA with Tukey's post hoc test (f, h). Synergy scores in (d, e) were calculated using the SynergyFinder ZIP model. ns, not significant; ^*^ *p* < 0.05; ^**^ *p* < 0.01; ^***^ *p* < 0.001; ^****^ *p* < 0.0001.

To evaluate the therapeutic potential of targeting the ID3‐SMAD3/NR2F2‐EPAS1 axis, we treated Osimertinib‐resistant NSCLC cells with SIS3, a phosphorylated‐SMAD3 inhibitor. This led to suppressed expression of both ID3 and NR2F2 and resulted in a dose‐dependent reduction in cell viability (Figure 7a,b). Similarly, treatment with Belzutifan, a second‐generation EPAS1 inhibitor, dose‐dependently reduced HIF2A protein levels (Figure S7j) and effectively inhibited the proliferation of resistant cells (Figure 7c). Notably, the combination of Osimertinib with either SIS3 or Belzutifan produced a pronounced synergistic effect, significantly enhancing growth suppression in Osimertinib‐resistant NSCLC cells (Figure 7d,e).

We further evaluated this combination in vivo using two independent Osimertinib‐resistant CDX models. In both H1975 AR and HCC827 AR xenografts, Osimertinib monotherapy showed minimal tumor growth inhibition, while Belzutifan monotherapy significantly suppressed tumor growth. The Osimertinib and Belzutifan combination produced a markedly stronger antitumor effect than either monotherapy, as reflected in tumor growth curves (Figure 7f), excised tumor images (Figure 7g), and final tumor weights (Figure 7h), without significant body weight loss across treatment groups (Figure S7k).

The following model (Figure 7i) depicts the super‐enhancer‐driven transcriptional circuit comprising ID3, SMAD3, and NR2F2, which critically sustains Osimertinib resistance in NSCLC. ID3 functions as a key co‐factor, directly interacting with SMAD3 and NR2F2, thereby enhancing their DNA‐binding affinity and potentiating oncogenic transcriptional programs. This circuit drives the expression of EPAS1 and GPX4/FTH1/NQO1, promoting neuroendocrine differentiation while suppressing ferroptosis. EPAS1 acts as a central integrator, linking neuroendocrine differentiation to ferroptosis inhibition, thus establishing a self‐reinforcing loop that maintains the resistant phenotype. Disruption of this ID3‐SMAD3/NR2F2‐EPAS1 axis restores Osimertinib sensitivity, through targeted inhibition with SIS3 and Belzutifan, positioning this pathway as a promising therapeutic target for overcoming Osimertinib resistance in NSCLC.

## 3 Discussion

Therapeutic resistance remains a major obstacle in cancer treatment, often driven by the dynamic remodeling of super‐enhancer (SE) landscapes [19, 30]. While the classical Core Regulatory Circuitry (CRC) model posits that a set of master transcription factors mutually reinforce expression through direct DNA binding, our findings delineate a structurally distinct, non‐canonical architectural paradigm underlying Osimertinib resistance. High SMAD3 expression predicts poor prognosis in lung cancer, and contributes to malignant progression [38], radioresistance [39], and resistance to immunotherapy through the activation of downstream signaling pathways [40]. NR2F2, notably in brain metastases, confers chemoresistance by activating GPX4 to inhibit ferroptosis [41]. ID3 exhibits dual functions: acting as a transcriptional repressor by sequestering bHLH factors [42, 43] or promoting immune evasion through MYC‐dependent PD‐L1 upregulation [44]. Our data reveal that ID3 operates through conformational reprogramming: by allosterically engaging the SMAD3‐NR2F2 complex, it stabilizes their retention on SEs and amplifies their transcriptional output. ID3 challenges the traditional CRC model by acting as a structural scaffold rather than an independent DNA binder. The identification of this circuit highlights a novel regulatory paradigm in which SEs activity coordinates both lineage‐related and stress‐responsive transcription factors, underscoring the complexity and adaptability of transcriptional circuitry underlying therapeutic resistance. Notably, treatment with the phosphorylated‐SMAD3 inhibitor SIS3 resulted in a marked suppression of ID3 and NR2F2 expression and effectively reduced the survival of Osimertinib‐resistant cells.

A notable feature of our findings is that individual CRC components were broadly upregulated in post‐treatment patient samples, yet many of these patients maintained clinical sensitivity to EGFR‐TKIs for prolonged periods. This apparent paradox is best reconciled through the “resistance continuum” framework, in which resistance develops through progressive cell‐state transitions and stepwise assembly of gene expression programs rather than through a binary switch [35]. The initial upregulation of ID3, SMAD3, and NR2F2 under therapeutic pressure likely reflects an early adaptive response associated with drug‐tolerant persister states [45], whereas the establishment of a fully functional, self‐reinforcing CRC requires sustained drug exposure and progressive epigenetic reinforcement. The median progression‐free survival of approximately 18.9 months with first‐line Osimertinib [5] may therefore reflect the time required for this transition. This interpretation is consistent with the broader view that non‐genetic mechanisms, including transcriptional reprogramming and chromatin remodeling, act as independent drivers of therapeutic resistance, with drug‐tolerant persister cells serving as a reservoir from which stable resistance subsequently evolves [46].

Neuroendocrine differentiation characterizes malignancies like small‐cell lung cancer and pancreatic neuroendocrine tumors, driving tumor plasticity and progression via metabolic reprogramming [47, 48]. Concurrently, ferroptosis is an iron‐dependent cell death defined by lethal lipid peroxidation and subsequent membrane disruption [49]. Interestingly, neuroendocrine pathways frequently intersect with ferroptosis regulation. For example, ASCL1 couples neuroendocrine transdifferentiation with ferroptosis resistance in prostate cancer [50], while MEN1 modulates ferroptosis sensitivity via PUFA metabolism in pancreatic neuroendocrine tumors [51]. Our study bridges these two pivotal processes by demonstrating, for the first time, that Osimertinib‐resistant NSCLC exhibits neuroendocrine differentiation features orchestrated by the ID3‐SMAD3/NR2F2 circuit. Simultaneously, this circuit suppresses ferroptosis, thereby fostering a multi‐layered resistance mechanism. We further identified EPAS1 (encoding HIF2A) as a critical downstream effector integrated within this regulatory circuit. Under the coordinated control of ID3, SMAD3, and NR2F2, EPAS1 simultaneously drives neuroendocrine differentiation and inhibits ferroptosis. Crucially, EPAS1 acts as a central hub orchestrating the bidirectional crosstalk between ferroptosis suppression and neuroendocrine differentiation to sustain the resistant phenotype.

Notably, EPAS1 gain‐of‐function mutations are associated with neuroendocrine tumors, including pheochromocytoma [52]. HIF2A, its protein product, functions as a ferroptosis‐related gene and plays oncogenic roles in pancreatic [53] and prostate cancers [54]. Importantly, Belzutifan [55], a second‐generation HIF2A inhibitor originally approved for the treatment of VHL‐associated tumors and currently under clinical investigation for castration‐resistant prostate cancer, renal cell carcinoma, and breast cancer (NCT04924075, NCT02861573, NCT06428396), exhibited marked anti‐proliferative effects in Osimertinib‐resistant NSCLC cells. Moreover, its combination with Osimertinib yielded synergistic anti‐tumor effects, supporting HIF2A inhibition as a promising strategy to counteract resistance in NSCLC.

## 4 Conclusion

In summary, our findings define a resistance mechanism where ID3 functions as a non‐canonical structural co‐factor. By stabilizing the SMAD3‐NR2F2 complex on chromatin, ID3‐SMAD3/NR2F2 potentiates a HIF2A‐driven program that couples neuroendocrine differentiation with ferroptosis evasion. These findings elucidate a previously unrecognized resistance mechanism and nominate the ID3‐SMAD3/NR2F2‐EPAS1 axis as a potential therapeutic target for overcoming resistance in NSCLC.

## 5 Methods

### 5.1 Cell Lines and Cell Culture

The human NSCLC cell lines HCC827, H1975, and PC9, as well as the 293T cell line, were obtained from the American Type Culture Collection (ATCC). Osimertinib‐resistant variants (designated HCC827 AR, H1975 AR, and PC9 AR) were established by continuous exposure of the corresponding parental lines to escalating concentrations of Osimertinib, ranging from 10 nm to 500 nmm. To maintain the resistant phenotype, 500 n Osimertinib was routinely supplemented in the culture medium. HCC827/AR, PC9/AR, and H1975/AR cells were maintained in RPMI 1640 medium (Gibco), and 293T cells were cultured in DMEM (Gibco). All cells were maintained in a humidified incubator at 37°C with 5% CO_2_.

### 5.2 Patient Samples

Tissue samples from EGFR‐TKI‐treated and treatment‐naïve patients were obtained from The First Affiliated Hospital, Sun Yat‐sen University (Guangzhou, Guangdong, China). This study was approved by the Medical Research Ethics Committee (Approval No. [2024]765), and written informed consent was obtained from all participating patients. This study does not involve any prospective clinical trial or interventional study; therefore, no clinical trial registration number is applicable.

### 5.3 Antibodies and Reagents

The following antibodies were used in this study: anti‐FLAG (#14793), Rabbit (DA1E) mAb IgG XP Isotype Control (#3900), and Mouse (G3A1) mAb IgG1 Isotype Control (#5415) were purchased from Cell Signaling Technology (CST). Anti‐SYP (17785‐1‐AP), anti‐CGA (23342‐1‐AP), and anti‐INSM1 (55477‐1‐AP) were obtained from Proteintech (Rosemont, USA). Anti‐SMAD3 (ab40854), anti‐NR2F2 (ab211777), anti‐HIF2A (ab243861), and anti‐H3K27ac (ab4729) were purchased from Abcam (Cambridge, MA). Anti‐ID3 (ER62074) was purchased from HUABIO (Zhejiang, China). Osimertinib (S7297) and Belzutifan (S8886) were obtained from Selleck Chemicals, dissolved in DMSO, and stored at −20°C. The final DMSO concentration was limited to 0.3% in all experiments.

### 5.4 MTT Cell Proliferation Assay

Cells were plated into 96‐well plates at densities ranging from 1,000 to 3,000 cells per well, depending on the experimental design, and maintained for the specified durations. At each measurement time point, MTT reagent (3‐(4,5‐dimethylthiazol‐2‐yl)‐2,5‐diphenyl tetrazolium bromide) was added to a final volume of 20 µL per well, and the plates were returned to 37°C for an additional 2 h to allow formazan formation. The resulting signal was read at 490 nm on a microplate spectrophotometer. For drug sensitivity assays, cells were exposed to a range of Osimertinib, SIS3, or Belzutifan concentrations for 48 h before MTT measurement.

### 5.5 Cell Migration Assay

For migration assays, 5 × 10^4^ cells suspended in serum‐free medium were loaded into the upper compartment of Transwell inserts with 8 µm pores, while the lower compartment was filled with FBS‐supplemented medium to provide a chemoattractant gradient. After 48 h, non‐migrated cells on the upper side of the membrane were gently removed, and cells that had transmigrated to the lower surface were fixed in 4% paraformaldehyde and visualized by crystal violet staining. Stained membranes were imaged under an inverted microscope, and migrated cell numbers were quantified with ImageJ software.

### 5.6 RT‐qPCR

Total RNA was extracted using the RNeasy Mini Kit (Qiagen), and purified RNA was reverse‐transcribed into cDNA using the Hifair III first Strand cDNA Synthesis SuperMix for qPCR (Yeasen, China). Quantitative real‐time PCR (qPCR) was performed on a CFX96 Real‐Time PCR Detection System (Bio‐Rad, Hercules, CA, USA) using Hieff qPCR SYBR Green Master Mix (Yeasen, China). Relative gene expression levels were normalized to GAPDH using the 2^‐ΔΔ^Ct method.

### 5.7 IP and Western Blotting

Cells were lysed on ice in RIPA buffer freshly supplemented with cocktails of protease and phosphatase inhibitors, and the resulting whole‐cell extracts were cleared by centrifugation. Equal amounts of total protein were separated on SDS‐PAGE gels and transferred onto PVDF membranes, which were then incubated with the indicated primary antibodies and detected with the corresponding HRP‐conjugated secondary antibodies. For immunoprecipitation, cleared lysates were rotated overnight at 4°C with anti‐FLAG M2 agarose beads (Sigma) to enrich FLAG‐tagged proteins and their associated complexes.

### 5.8 IHC and Immunofluorescence

Tissue sections and cultured cells were incubated with primary antibodies overnight at 4°C. For immunohistochemistry (IHC), signal amplification was performed using the ABC peroxidase kit (Boster), followed by visualization with DAB. For immunofluorescence (IF), cells on coverslips were incubated with fluorescence‐conjugated secondary antibodies (Abcam) for 1 h, counterstained with DAPI, and mounted for imaging.

### 5.9 Expression Plasmids, shRNA, Small Interfering RNA (siRNA), and sgRNA

Full‐length human cDNA sequences encoding ID3, SMAD3, NR2F2, and EPAS1 were cloned into the pCDH‐CMV‐MCS‐EF1‐Puro vector with either FLAG or HA tags. shRNA constructs were cloned into the pLKO.1‐Puro vector (Addgene), and sgRNA constructs were generated using the pLV hU6‐sgRNA hUbC‐dCas9‐KRAB‐T2a‐Puro backbone.

### 5.10 Dual Luciferase Reporter Assay

Candidate cis‐regulatory regions, including selected promoter and enhancer elements, were PCR‐amplified from human genomic DNA and cloned into the pGL3‐Basic or pGL3‐Promoter vector immediately upstream of the firefly luciferase coding sequence. Each construct was co‐introduced into cells together with a Renilla luciferase plasmid, which provided an internal reference for normalization of transfection efficiency. After a 48‐h incubation, cells were harvested and lysed, and the activities of firefly and Renilla luciferases in the same lysate were measured sequentially with the Dual‐Luciferase Reporter Assay System. Promoter or enhancer activity was reported as the ratio of firefly‐to‐Renilla luminescence.

### 5.11 Lipid ROS Detection

Cells were collected and resuspended in serum‐free medium containing the C11‐BODIPY 581/591 fluorescent probe (Thermo Fisher Scientific). After incubation for 30 min at 37°C in the dark, cells were washed to remove unincorporated probe and immediately analyzed by flow cytometry. The shift in emission of the oxidized form of C11‐BODIPY was detected in the FITC channel, and the mean fluorescence intensity was used to assess intracellular lipid peroxidation levels.

### 5.12 Ferrous Ion (Fe^2^^+^) Detection

Cells were seeded and stained with 5 µm FerroOrange probe (Dojindo) in serum‐free medium for 30 min at 37°C in the dark. Live‐cell imaging was then performed on a laser scanning confocal microscope, with excitation at 561 nm.

### 5.13 Soft Agar Colony Formation Assay

Cells were trypsinized, counted, and suspended in complete medium containing 0.3% agar at a density of 500 cells per well of a 6‐well plate. The plates were maintained at 37°C with 5% CO_2_ for 2 to 3 weeks, with periodic addition of fresh medium to prevent drying. At the endpoint, colonies were stained, imaged, and quantified.

### 5.14 Colony Formation Assay

Cells were seeded at low density and allowed to adhere overnight. For knockdown experiments, cells were stably transduced with PLKO control or shRNA targeting *SMAD3*, *NR2F2*, or *ID3* prior to seeding; for overexpression experiments, cells were transduced with PCDH control or PCDH‐FLAG‐tagged constructs. The following day, cells were treated with vehicle (DMSO) or the indicated concentration of Osimertinib, and cultured for 10–14 days, with medium replaced every 3 days. At the endpoint, colonies were washed twice with PBS, fixed with 4% paraformaldehyde for 15 min at room temperature, and stained with 0.1% crystal violet solution for 20 min. Excess dye was removed by gentle washing with distilled water, and plates were air‐dried before imaging. Colony number and area were quantified using ImageJ software (NIH). Each experiment was performed with three biological replicates.

### 5.15 Long‐term Osimertinib Escape Assay

Parental H1975 and HCC827 cells stably expressing PLKO, shSMAD3, shNR2F2, or shID3 were exposed to stepwise‐escalating concentrations of Osimertinib (10 nM, 25 nM, 50 nM, and 100 nM) over 8 weeks, with each concentration maintained for 2 weeks. Drug‐containing medium was refreshed every 2–3 days. IC_50_ values were determined at the start and end of selection by treating cells with serial dilutions of Osimertinib for 72 h, followed by MTT assay, and IC_50_ fold change was calculated relative to Day 0. In parallel, colony formation assays were performed by seeding cells at low density into 6‐well plates and culturing them in the presence or absence of Osimertinib for 10–14 days; colonies were fixed with 4% paraformaldehyde, stained with 0.1% crystal violet, and quantified using ImageJ. The same colony formation procedure was used to assess the effect of FLAG‐SMAD3, FLAG‐NR2F2, or FLAG‐ID3 overexpression on Osimertinib tolerance in parental HCC827 cells.

### 5.16 Drug Combination Analysis

Cells were exposed to a dose matrix covering a range of concentrations for each compound, and viability was measured 72 h after treatment. The resulting response surface was analyzed under the Zero Interaction Potency (ZIP) framework, with synergy scores computed using the SynergyFinder web tool.

### 5.17 Xenograft Assays in Nude Mice

All in vivo procedures were carried out under a protocol reviewed and approved by the Institutional Animal Care and Use Committee of SHANGHAI MODEL ORGANISMS (Shanghai, China; Approval No. GY2024‐803), in accordance with relevant ethical guidelines. Four‐week‐old nude mice received subcutaneous inoculations of tumor cells from the indicated groups, including H1975 AR cells stably expressing PLKO, shID3, shSMAD3, or shNR2F2, as well as parental H1975 cells. When tumor xenografts grew to approximately 150 mm^3^, mice were randomly assigned to receive either Osimertinib, Belzutifan, the Osimertinib+Belzutifan combination, or CMC‐Na vehicle. Tumor dimensions were recorded at regular intervals throughout the treatment period, and animals were euthanized once tumor volume reached the predefined humane endpoint of 1500 mm^3^.

### 5.18 WES Analysis

All alignments were performed using the hg19 reference genome by BWA (version 0.7.17) with default parameters [56]. Mapped reads were sorted by SAMtools (version 1.13), and PCR duplicates were removed with the Picard MarkDuplicates tool (version 1.136). Variants were called using DRIVER tools [https://www.biorxiv.org/content/10.1101/115717v2.full.pdf] and filtered using the options “DP < 5.0 || QD < 2.0 || FS > 60.0 || MQ < 40.0 || SOR > 3.0 || MQRankSum < −12.5 || ReadPosRankSum < −8.0”.

### 5.19 ChIP‐Seq and CUT&Tag Analysis

ChIP uses the method described before, and CUT&Tag was performed according to the manufacturer's instructions (TD903, Vazyme). The tumor tissue was completely crushed by using a homogenizer, and then centrifugally filtered under a 25 µm filter. The obtained filtrate can continue to be carried out according to the CUT&Tag step.

Sequencing reads were first aligned to the hg19 human reference genome using BWA (v0.7.17) under default settings56. The resulting alignments were filtered for high mapping quality and sorted with SAMtools (v1.13)57. PCR duplicates were subsequently identified and discarded using Picard MarkDuplicates (v1.136), after which reads overlapping ENCODE blacklist regions were excluded using BEDTools (v2.30.0).

For histone ChIP‐seq analysis, peaks were called using MACS2 (Model‐Based Analysis of ChIP‐Seq, version 2.2.9.1) with the parameters “‐q 0.01 ‐extsize = 146 ‐nomodel ‐B” [57]. Bigwig files were created using the deepTools bamCompare function (version 3.1.3) with the settings “‐operation subtract ‐normalizeUsing CPM ‐extendReads 146 ‐binSize 20” [58]. For Cut‐tag data, peaks were identified with MACS2 using default options, and Bigwig files were generated using the deepTools bamCoverage function with standard settings.

### 5.20 SE Identification

SE identification for each cell line was performed using the Rank Order of Super Enhancers (ROSE) tool [17]. In this process, ROSE merged enhancer elements within a 12 kb range and ranked them based on their intensity. SEs were defined as stitched elements exhibiting a tangent slope with an inflection point value of ≥1.

### 5.21 RNA‐Seq Analysis

Raw reads were aligned to the GRCh37 reference genome utilizing HISAT2 (version 2.2.1)^59^and quantified with the htseq‐count program (version 2.0.8) under default settings. The raw read counts were then normalized using FPKM. Differentially expressed genes were determined through the DESeq2 package (version 1.42.1) [60], applying a threshold of adjusted *p*‐value < 0.05 and an absolute log2 fold‐change (shRNA vs. control) greater than 0.5.

### 5.22 Single‐Cell RNA‐Seq Analysis

We obtained single‐cell transcriptomic data of Osimertinib‐treated lung cancer cell line (PC9) from the public repository GSE247684. Raw count matrices were loaded and processed using Seurat (v5.1.0). Data normalization was performed via the global‐scaling “LogNormalize” method with a scale factor of 10 000. The top 2 000 highly variable genes (HVGs) were identified using FindVariableFeatures for dimensionality reduction. Comprehensive gene expression standardization was applied by scaling all detected genes via the ScaleData function. Dimensionality reduction was conducted through principal component analysis (PCA) using these HVGs. Cell clustering was performed by constructing a shared nearest‐neighbor (SNN) graph followed by Louvain algorithm‐based community detection (FindNeighbors and FindClusters), with cluster visualization achieved via uniform manifold approximation and projection (UMAP) embedding.

### 5.23 TF Co‐Binding Analysis

Genomic intervals showing overlap between H3K27ac, H3K4me1, or H3K4me3 signal and the binding peaks of the three transcription factors were extracted with the intersect function of BEDTools. Promoter regions were defined as loci co‐marked by H3K27ac and H3K4me3, while enhancers were defined as loci co‐marked by H3K27ac and H3K4me1. Within each category, regulatory elements were further stratified according to TF occupancy into three groups: “Solo” (occupied by a single TF), “Dual” (occupied by any two TFs), and “Trio” (occupied by all three TFs simultaneously).

#### 5.23.1 Gene set enrichment analysis (GSEA)

For each transcription factor, genes that exhibited significantly reduced expression upon its individual knockdown were assembled into a custom gene set. Ranked gene lists were then generated based on fold‐change values calculated between each knockdown sample and its corresponding control, and these rankings were used as the input for the GSEAPreranked algorithm.

### 5.24 Molecular Dynamics Simulation

3D structural models of ID3, SMAD3, and NR2F2 were generated de novo using AlphaFold2 based on their protein sequences. The double‐helix structure of the NR2F2 enhancer was modeled using Discovery Studio Visualizer (v24.1.0.23298). Protein–protein and protein–DNA docking was performed using HDOCK, and the top‐scoring structure was selected for subsequent interaction analysis.

All molecular dynamics simulations were performed in GROMACS 2022.1. Each system was thermally equilibrated at 298.15 K using the V‐rescale thermostat, while pressure was held at 1.0 bar by the Parrinello–Rahman barostat. Both proteins and DNA were modeled with the Amber99SB all‐atom force field, and water molecules were represented using the TIP3P model. 3D periodic boundary conditions were applied throughout the simulations. Trajectory analyses were carried out with the built‐in tools provided by GROMACS. Molecular structures were rendered in PyMOL (http://www.pymol.org/), and quantitative results were plotted with the Matplotlib library in Python.

### 5.25 Statistical Analysis

Data are presented as mean ± standard deviation (SD) from at least three independent biological replicates. Statistical analyses were performed using GraphPad Prism 10.0. Two‐tailed Student's *t*‐test was used for two‐group comparisons, and one‐way or two‐way ANOVA followed by Tukey's multiple comparisons test was used for multi‐group comparisons. *p* < 0.05 was considered statistically significant.

## Author Contributions

A.C.L., Z.G.L., and L.Z.J. contributed equally to this work as co‐first authors. X.P.S., Y.Y.Z., W.J.M., and W.Y. conceived and directed the study as co‐corresponding authors. A.C.L., Z.G.L., X.Y.L., Q.P., and L.Z.J. performed the experiments. A.D.H., B.Y.L., S.W.H., and J.J.H. analyzed and interpreted the data. A.D.H. and L.Z.J. provided bioinformatics analysis. Z.G.L., X.Q.L., W.J.M., and L.Y.Y. were responsible for providing clinical advice. A.C.L. wrote the first draft of the manuscript. X.P.S., L.L.J., W.Y., and Y.Y.Z. revised the manuscript. All the authors had full access to the data and reviewed the manuscript before submission.

## Conflicts of Interest

The authors declare no conflict of interest.

## Supporting information




**Supporting File**: advs75765‐sup‐0001‐SuppMat.docx.

## Data Availability

The data that support the findings of this study are openly available in GEO datasets at https://www.ncbi.nlm.nih.gov/geo/, reference numbers GSE308413, GSE308412, and GSE308411. The public data analysed in this project include RNA‐Seq (GSE247684, GSE253742, GSE231938, GSE243569) and ChIP‐Seq (PRJNA834760).
