# Supplementary material for: A Non‐Canonical Core Transcriptional Regulatory Circuit Orchestrates Chromatin Reprogramming to Drive Osimertinib Resistance in Non‐Small Cell Lung Cancer
Source: Adv Sci (Weinh). 2026 May 20;13(46):e75765. doi: 10.1002/advs.75765 (PMC13335962; doi:10.1002/advs.75765)
Supplement: Supplementary file 1 [file ADVS-13-e75765-s001.docx]

**Supplementary Data Fig. 1 Differential Characteristics Between Osimertinib-Resistant and -Sensitive Cell Lines. (a)** Bright-field morphology of three paired parental and Osimertinib-resistant NSCLC cell lines (H1975/H1975 AR, HCC827/HCC827 AR, PC9/PC9 AR). **(b)** Immunofluorescence staining of the neuroendocrine marker SYN (green) in HCC827 AR and parental HCC827 cells; DAPI (blue) marks nuclei. **(c)** Immunofluorescence staining of the neuroendocrine marker MAP2 (green) in PC9 AR, PC9, HCC827 AR, and HCC827 cells; DAPI (blue) marks nuclei. **(d)** Circos plots displaying the genome-wide distribution of exonic mutations specific to each Osimertinib-resistant cell line (H1975 AR-specific, HCC827 AR-specific, PC9 AR-specific) compared with their parental counterparts, identified by whole-exome sequencing. **(e)** Heatmaps of exonic mutations in genes belonging to the EGFR, EGFR-TKI tolerance, KRAS, and TP53 pathways across the three cell line pairs; mutation types are color-coded as indicated. **(f)** GO Biological Process enrichment of differentially gained H3K27ac ChIP-seq peaks in Osimertinib-resistant cells (left) and parental cells (right). **(g)** GO Biological Process enrichment of upregulated genes from RNA-seq in Osimertinib-resistant cells (left) and parental cells (right), shown separately for H1975, HCC827, and PC9 pairs. Dot size represents gene count and color indicates adjusted *P*-value.

GO Biological Process enrichment in (f, g) was performed using Metascape, with significance assessed by hypergeometric test and Benjamini–Hochberg correction for multiple testing; adjusted *P*-values are shown by the color scale and gene counts by dot size.

**Supplementary Data Fig. 2 Super-Enhancers Drive TF-Mediated Osimertinib Resistance in NSCLC. (a)** UMAP visualization of single-cell RNA-seq data from PC9 cells under different Osimertinib treatment conditions (GSE247684). C, untreated parental control; P1.2, drug-tolerant persister cells generated by acute treatment with 1.2 μM Osimertinib for 9 days; T0008–T1.2, cell populations adapted to progressively increasing Osimertinib concentrations through stepwise dose escalation (T0008 = 8 nM; T0016 = 16 nM; T0032 = 32 nM; T0075 = 75 nM; T0150 = 150 nM; T0300 = 300 nM; T0600 = 600 nM; T1.2 = 1.2 μM). **(b)** Box plot comparing the combined enrichment score of ID3, SMAD3, and NR2F2 between non-treatment and EGFR-TKI-treated NSCLC samples (GSE243569). **(c)** IGV tracks showing H3K27ac ChIP-seq signals at the *SMAD3*, *NR2F2*, and *ID3* loci in HCC827 AR, HCC827, H1975 AR, H1975, PC9 AR, and PC9 cell lines, together with patient-derived samples (treatment, *n* = 7; non-treatment, *n* = 3). H3K4me1 and H3K4me3 tracks from HCC827 AR are shown for reference. **(d)** Kaplan–Meier overall survival analysis of NSCLC patients in the TCGA cohort stratified by high versus low expression of *SMAD3*, *NR2F2*, and *ID3*. **(e)** Western blot validating shRNA-mediated knockdown of SMAD3, NR2F2, and ID3 in parental HCC827 cells. **(f)** Western blot validating shRNA-mediated knockdown of SMAD3, NR2F2, and ID3 in HCC827 AR, H1975 AR, and PC9 AR cells.

Statistical significance in (b) was assessed by two-tailed Wilcoxon rank-sum test; box plots show median, interquartile range, and 1.5× IQR whiskers. Statistical significance in (d) was assessed by log-rank test, with hazard ratios (HR) and *P*-values indicated.

**Supplementary Data Fig. 3 ID3 Enhances SMAD3/NR2F2 Binding to Activate Transcription. (a)** Left: ChIP-seq enrichment profiles of NR2F2, SMAD3, ID3, H3K27ac, H3K4me1, and H3K4me3 at the *ID3* locus in HCC827 AR cells, showing two candidate cis-regulatory elements (ID3-CE1, ID3-CE2) and the *ID3* promoter (ID3-P). Right: schematic of ID3-CE1 (chr1: 23,801,782–23,801,916) showing the predicted binding motifs for SMAD3, NR2F2, and ID3. **(b)** Left: ChIP-seq enrichment profiles at the *SMAD3* locus in HCC827 AR cells, showing the *SMAD3* promoter (SMAD3-P) and two candidate cis-regulatory elements (SMAD3-CE1, SMAD3-CE2). Right: schematic of SMAD3-CE1 (chr15: 67,441,574–67,441,678) showing the predicted binding motifs for NR2F2, SMAD3, and ID3. **(c)** Luciferase reporter assay measuring SMAD3 promoter activity in HCC827 AR cells following shRNA-mediated knockdown of SMAD3, NR2F2, or ID3. **(d)** Luciferase reporter assays measuring SMAD3-CE1 and SMAD3-CE2 activity in HCC827 AR cells under the same knockdown conditions as in (c). **(e)** Luciferase reporter assay measuring ID3 promoter (ID3-P) activity in HCC827 AR cells following knockdown of SMAD3, NR2F2, or ID3. **(f)** Luciferase reporter assays measuring ID3-CE1 and ID3-CE2 activity in HCC827 AR cells under the same knockdown conditions as in (e). **(g)** qPCR analysis of *ID3*, *SMAD3*, and *NR2F2* mRNA levels in HCC827 AR cells following KRAB–dCas9-mediated repression of the ID3 enhancer ID3-CE1. **(h)** Cell viability of HCC827 AR cells with ID3-CE1 repression. **(i)** Dose-response curves of HCC827 AR cells with ID3-CE1 repression after Osimertinib treatment. **(j)** ChIP-qPCR showing FLAG-SMAD3 and FLAG-NR2F2 occupancy at the SMAD3, NR2F2, and ID3 promoters in HCC827 AR cells, with or without co-expression of HA-ID3. **(k)** Root-mean-square deviation (RMSD) of NR2F2–DNA versus ID3–NR2F2–DNA (top) and SMAD3–DNA versus ID3–SMAD3–DNA (bottom) complexes over 100 ns molecular dynamics (MD) simulations. **(l)** Root-mean-square fluctuation (RMSF) per residue for the same complexes as in (k). **(m)** Representative structural snapshots of NR2F2–DNA, ID3–NR2F2–DNA, SMAD3–DNA, and ID3–SMAD3–DNA complexes at 0 ns and 100 ns of MD simulation. **(n)** Total interaction energy and its decomposition into short-range Lennard-Jones (LJ-SR) and Coulomb (Coulomb-SR) components for the four complexes over the simulation trajectory. **(o)** Principal component analysis (PCA) of MD trajectories for NR2F2–DNA, ID3–NR2F2–DNA, SMAD3–DNA, and ID3–SMAD3–DNA complexes. Each data point represents an individual conformational snapshot, colored from blue (early phase) through white (intermediate phase) to red (late phase) of the simulation. Scree plots (lower right of each panel pair) show the proportion of variance explained by each principal component.

Data in (c–g, j) are presented as mean ± SD from *n* = 3 independent experiments. Data in (h, i) are presented as mean ± SD from *n* = 3 technical replicates. Statistical significance was assessed by one-way ANOVA with Dunnett's post hoc test versus PLKO/CTRL (c–g), two-way ANOVA with Tukey's post hoc test (h, i), or two-tailed unpaired Student's *t*-test (j). ns, not significant; **P* < 0.05; ***P* < 0.01; ****P* < 0.001; *****P* < 0.0001.

**Supplementary Data Fig. 4 ID3, SMAD3, and NR2F2 Synergistically Regulate Resistance Programs.** **(a)** Volcano plots of differentially expressed genes following shSMAD3 (left), shNR2F2 (middle), or shID3 (right) versus PLKO control in HCC827 AR cells. **(b)** Reciprocal GSEA showing enrichment of up-regulated genes from each knockdown (shNR2F2, shID3, shSMAD3) in the ranked gene lists of the other two knockdowns in HCC827 AR cells. **(c)** GSEA showing enrichment of the SMAD3/NR2F2/ID3 co-down-regulated gene set in genes ranked by differential expression between H1975 resistant versus sensitive cells (top) and PC9 resistant versus sensitive cells (bottom). **(d)** GO Biological Process, KEGG, and Reactome pathway enrichment of up-regulated genes in HCC827 AR versus HCC827 and H1975 AR versus H1975. **(e)** Western blot of total EGFR and phosphorylated EGFR (p-EGFR) in parental and Osimertinib-resistant HCC827, PC9, and H1975 cell lines treated with vehicle (0) or Osimertinib (0.25, 0.75 μM).

GSEA in (b, c) was performed using the GSEA preranked algorithm; normalized enrichment scores and nominal *P*-values are shown. Pathway enrichment in (d) was assessed by hypergeometric test with Benjamini–Hochberg correction; *q*-values and gene counts are shown by the color and size scales, respectively.

**Supplementary Data Fig. 5 ID3, SMAD3, and NR2F2 Drive Malignancy in Osimertinib-Resistant NSCLC.** **(a)** Heatmaps showing knockdown efficiency of *SMAD3*, *NR2F2*, and *ID3* by qPCR in H1975 AR, HCC827 AR, and PC9 AR cells transfected with two independent siRNAs targeting each gene. **(b)** Western blot validating FLAG-tagged ID3, NR2F2, and SMAD3 overexpression in parental HCC827, H1975, and PC9 cells. **(c)** Transwell migration assays in HCC827, H1975, and PC9 cells overexpressing FLAG-SMAD3, FLAG-NR2F2, or FLAG-ID3; representative images (top) and quantification relative to PCDH control (bottom). **(d)** Heatmaps showing percent cell viability of HCC827, H1975, and PC9 cells overexpressing FLAG-SMAD3, FLAG-NR2F2, or FLAG-ID3 across a range of Osimertinib concentrations. **(e)** Tumor weights at endpoint from subcutaneous xenografts in nude mice using H1975 AR cells stably transduced with PLKO, shSMAD3, shNR2F2, or shID3, treated with CMC-Na vehicle or Osimertinib, alongside parental H1975 xenografts treated with CMC-Na or Osimertinib. **(f)** Western blot of SMAD3, NR2F2, and ID3 in HCC827 AR cells following single or double shRNA-mediated knockdown of these factors. **(g)** Cell viability of HCC827 AR cells following single or combined knockdown of the indicated factors.

Data in (a, c, g) are presented as mean ± SD from *n* = 3 independent experiments. Data in (d) are presented as mean ± SD from *n* = 3 technical replicates. Data in (e) are presented as mean ± SD (*n* = 6 mice per group). Statistical significance was assessed by one-way ANOVA with Dunnett's post hoc test versus NC/PCDH (a, c), two-tailed unpaired Student's *t*-test versus PCDH at each Osimertinib concentration (d), one-way ANOVA with Tukey's post hoc test (e), or two-way ANOVA with Tukey's post hoc test (g). **P* < 0.05; ***P* < 0.01; ****P* < 0.001; *****P* < 0.0001.

**Supplementary Data Fig. 6** **ID3, SMAD3, and NR2F2 Are Required for the Acquisition of Osimertinib Resistance.(a)** Colony formation assays in parental H1975 (left) and HCC827 (right) cells stably transduced with PLKO, shSMAD3, shNR2F2, or shID3, cultured in the absence (–) or presence (+) of Osimertinib; representative images (top) and quantification of relative colony area (bottom). **(b)** Stepwise Osimertinib dose escalation in H1975 (left) and HCC827 (right) cells transduced with PLKO, shSMAD3, shNR2F2, or shID3 over 8 weeks. The dual x-axis indicates the Osimertinib concentration applied during escalation (top, nM) and the corresponding escalation time (bottom, weeks); the y-axis shows IC_50_ fold change relative to week 0. **(c)** Osimertinib dose-response curves of H1975 (left) and HCC827 (right) cells transduced with PLKO, shSMAD3, shNR2F2, or shID3 at the end of dose escalation. **(d)** Colony formation assays in HCC827 cells overexpressing FLAG-SMAD3 (SMAD3-OE), FLAG-NR2F2 (NR2F2-OE), or FLAG-ID3 (ID3-OE) in the absence (–) or presence (+) of Osimertinib. Representative images (left) and quantification of relative colony area (right).

Data are presented as mean ± SD from *n* = 3 independent experiments. Statistical significance was assessed by two-way ANOVA with Tukey's post hoc test (a, d), or two-way ANOVA with Tukey's post hoc test across the time course (b). **P* < 0.05; ***P* < 0.01; ****P* < 0.001; *****P* < 0.0001.

**Supplementary Data Fig. 7 Neuroendocrine and Ferroptosis Interaction Drives Osimertinib Resistance in NSCLC.** **(a)** Western blot of CGA and SYP in H1975 AR cells following shRNA-mediated knockdown of SMAD3, NR2F2, or ID3 with two independent shRNAs. **(b)** Immunofluorescence staining of MAP2 (green) in HCC827 AR cells following knockdown of SMAD3, NR2F2, or ID3; DAPI (blue) marks nuclei. **(c)** Immunofluorescence staining of MAP2 in parental H1975 cells overexpressing FLAG-SMAD3, FLAG-NR2F2, or FLAG-ID3. **(d)** Pearson correlation between *EPAS1* expression and the combined ID3/SMAD3/NR2F2 signature in EGFR-TKI-untreated NSCLC patient samples. **(e)** Dot plot showing *EPAS1* expression stratified by MAP2-high versus MAP2-low PC9 cell populations across increasing Osimertinib treatment conditions, from single-cell RNA-seq data (GSE247684). **(f)** Cell viability of H1975 AR cells with EPAS1 knockdown. **(g)** Osimertinib dose-response curves of H1975 AR cells with EPAS1 knockdown. **(h)** Cell viability of HCC827 AR cells with shSMAD3, shNR2F2, or shID3, treated with the indicated Osimertinib concentrations (0, 6.25, 12.5 μM) in the absence or presence of Ferrostatin-1 (Fer-1). **(i)** Immunofluorescence staining of MAP2 in HCC827 AR cells with shSMAD3, shNR2F2, or shID3, with or without Fer-1 treatment. **(j)** Western blot of HIF2A in HCC827 AR, H1975 AR, and PC9 AR cells treated with the indicated concentrations of Belzutifan. **(k)** Body weights of nude mice bearing H1975 AR-CDX (top) and HCC827 AR-CDX (bottom) treated with CMC-Na vehicle, Osimertinib, Belzutifan, or the Osimertinib+Belzutifan combination (Combo) over the treatment period.

Data in (f, g, h) are presented as mean ± SD from *n* = 3 independent experiments. Data in (k) are presented as mean ± SEM (*n* = 6 mice per group). Statistical significance was assessed by two-way ANOVA with Tukey's post hoc test (f, g, h, k), or two-tailed Pearson correlation (d). **P* < 0.05; ***P* < 0.01; ****P* < 0.001; *****P* < 0.0001.

**Supplementary Data Table 1** Source of Tissue Samples.

| ID | Gender | Pathological type | EGFR status | Biopsy/Resection | Pre-Biopsy/Resection Treatment History | Clinical Course |
| --- | --- | --- | --- | --- | --- | --- |
| P1 | Female | Lung adenocarcinoma | Exon 21 L858R | Resection | ­ | Postoperative adjuvant Almonertinib therapy |
| P2 | Female | Lung adenocarcinoma | Exon 21 L858R | Resection | ­ | Postoperative adjuvant Osimertinib therapy |
| P3 | Female | Lung adenocarcinoma | Exon 19 del | Resection | ­ | Postoperative adjuvant Almonertinib therapy |
| P4 | Female | Lung adenocarcinoma | G719A/S768I | Resection | Osimertinib | Postoperative adjuvant durg therapy |
| P5 | Female | Lung adenocarcinoma | Exon 19 del | Resection | Osimertinib | Postoperative adjuvant durg therapy |
| P6 | Male | Lung adenocarcinoma | Exon 19 del | Resection | Osimertinib | Recurrence followed perioperative treatment |
| P7 | Female | Lung adenocarcinoma | Exon 19 del | Resection | Almonertinib | Recurrence followed perioperative treatment |
| P8 | Male | Lung adenocarcinoma | Exon 21 L858R | Biopsy | Afatinib; Osimertinib | Acquired drug resistance |
| P9 | Female | Lung adenocarcinoma | Exon 21 L858R | Biopsy | Osimertinib | Acquired drug resistance |
| P10 | Male | Lung adenocarcinoma | Exon 19 del | Biopsy | Furmonertinib | Acquired drug resistance |

**Supplementary Data Table 2** Molecular Interactions and Binding Site Analysis.

| Name | Category | Group |
| --- | --- | --- |
| NR2F2:ARG128:HH12 - DNA-A:DG24:O1P | Salt Bridge | NR2F2-DNA |
| NR2F2:ARG128:HH22 - DNA-A:DG24:O1P | Salt Bridge | NR2F2-DNA |
| NR2F2:ARG128:NH1 - DNA-A:DA25:O1P | Electrostatic | NR2F2-DNA |
| NR2F2:SER112:HN - DNA-B:DT1:O5' | Hydrogen Bond | NR2F2-DNA |
| NR2F2:THR157:HG1 - DNA-B:DC2:O2P | Hydrogen Bond | NR2F2-DNA |
| NR2F2:GLN158:HE21 - DNA-B:DA3:O2P | Hydrogen Bond | NR2F2-DNA |
| NR2F2:ARG116:HH12 - DNA-A:DT23:O2P | Salt Bridge | ID3-NR2F2-DNA |
| NR2F2:ARG116:HH21 - DNA-A:DG24:O1P | Salt Bridge | ID3-NR2F2-DNA |
| NR2F2:ARG116:NH1 - DNA-A:DG24:O2P | Electrostatic | ID3-NR2F2-DNA |
| NR2F2:ARG116:NH2 - DNA-A:DT23:O1P | Electrostatic | ID3-NR2F2-DNA |
| NR2F2:ARG109:HH21 - DNA-B:DT1:O2 | Hydrogen Bond | ID3-NR2F2-DNA |
| NR2F2:ASN110:HD21 - DNA-B:DT1:O5' | Hydrogen Bond | ID3-NR2F2-DNA |
| NR2F2:SER112:HG - DNA-B:DT1:O4 | Hydrogen Bond | ID3-NR2F2-DNA |
| NR2F2:THR114:HG1 - DNA-A:DG24:O1P | Hydrogen Bond | ID3-NR2F2-DNA |
| NR2F2:SER112:O - DNA-A:DA25:H61 | Hydrogen Bond | ID3-NR2F2-DNA |
| SMAD3:PRO10:HN2 - DNA-B:DA5:O2P | Salt Bridge | SMAD3-DNA |
| SMAD3:LYS13:HZ1 - DNA-B:DA4:O2P | Salt Bridge | SMAD3-DNA |
| SMAD3:LYS41:HZ3 - DNA-A:DC15:O1P | Salt Bridge | SMAD3-DNA |
| SMAD3:LYS13:NZ - DNA-B:DG3:O2P | Electrostatic | SMAD3-DNA |
| SMAD3:LYS33:NZ - DNA-A:DC17:O1P | Electrostatic | SMAD3-DNA |
| SMAD3:LYS40:NZ - DNA-B:DC7:O1P | Electrostatic | SMAD3-DNA |
| SMAD3:LYS41:NZ - DNA-A:DC14:O2P | Electrostatic | SMAD3-DNA |
| SMAD3:LYS43:NZ - DNA-B:DG6:O2P | Electrostatic | SMAD3-DNA |
| SMAD3:LYS44:NZ - DNA-A:DC14:O1P | Electrostatic | SMAD3-DNA |
| SMAD3:LYS81:NZ - DNA-B:DG15:O2P | Electrostatic | SMAD3-DNA |
| SMAD3:ILE11:HN - DNA-B:DA5:O2P | Hydrogen Bond | SMAD3-DNA |
| SMAD3:SER37:HG - DNA-A:DC15:O2P | Hydrogen Bond | SMAD3-DNA |
| SMAD3:SER78:HN - DNA-A:DC15:O2P | Hydrogen Bond | SMAD3-DNA |
| SMAD3:SER78:HG - DNA-A:DT16:O1P | Hydrogen Bond | SMAD3-DNA |
| SMAD3:PRO10:HN2 - DNA-B:DA4:O1P | Salt Bridge | ID3-SMAD3-DNA |
| SMAD3:LYS36:HZ3 - DNA-A:DC17:O1P | Salt Bridge | ID3-SMAD3-DNA |
| SMAD3:LYS40:HZ1 - DNA-A:DT16:O1P | Salt Bridge | ID3-SMAD3-DNA |
| SMAD3:LYS43:HZ2 - DNA-B:DA5:O2P | Salt Bridge | ID3-SMAD3-DNA |
| SMAD3:ARG74:HH22 - DNA-B:DG16:O2P | Salt Bridge | ID3-SMAD3-DNA |
| SMAD3:PRO10:N - DNA-B:DG3:O1PV | Electrostatic | ID3-SMAD3-DNA |
| SMAD3:ARG14:NH1 - DNA-B:DA4:O2P | Electrostatic | ID3-SMAD3-DNA |
| SMAD3:LYS33:NZ - DNA-B:DT13:O2P | Electrostatic | ID3-SMAD3-DNA |
| SMAD3:LYS40:NZ - DNA-A:DC15:O2P | Electrostatic | ID3-SMAD3-DNA |
| SMAD3:LYS41:NZ - DNA-A:DC15:O2P | Electrostatic | ID3-SMAD3-DNA |
| SMAD3:LYS41:NZ - DNA-A:DC15:O2P | Electrostatic | ID3-SMAD3-DNA |
| SMAD3:LYS44:NZ - DNA-B:DG6:O1P | Electrostatic | ID3-SMAD3-DNA |
| SMAD3:ARG74:NH1 - DNA-B:DG16:O1P | Electrostatic | ID3-SMAD3-DNA |
| SMAD3:LYS81:NZ - DNA-B:DG15:O2P | Electrostatic | ID3-SMAD3-DNA |
| SMAD3:ILE11:HN - DNA-B:DA4:O1P | Hydrogen Bond | ID3-SMAD3-DNA |
| SMAD3:LYS43:HZ3 - DNA-B:DA5:O5' | Hydrogen Bond | ID3-SMAD3-DNA |
| SMAD3:GLN76:HE21 - DNA-A:DC15:O4' | Hydrogen Bond | ID3-SMAD3-DNA |
| SMAD3:GLN76:HE22 - DNA-A:DC14:O2 | Hydrogen Bond | ID3-SMAD3-DNA |
| SMAD3:LYS81:HZ2 - DNA-B:DG15:O5' | Hydrogen Bond | ID3-SMAD3-DNA |
| SMAD3:LYS81:HZ2 - DNA-B:DG15:O4' | Hydrogen Bond | ID3-SMAD3-DNA |
